# A dual approach to evaluate the performance of RNA-Seq data analysis pipelines with weak signals

**DOI:** 10.1093/bioadv/vbag134

**Published:** 2026-05-09

**Authors:** Malek Baroudi, Fadoum Ousmane Ly, Elen Goujon, Chrystelle Ibanez, Léo Macé, Habib Zouali, Olivier Armant, Stéphane Grison, Mohamedamine Benadjaoud, Imène Garali

**Affiliations:** Autorité de Sûreté Nucléaire et de Radioprotection (ASNR), PSE-SANTE/SESANE/LRTOX, Fontenay aux Roses, F-92260, France; Autorité de Sûreté Nucléaire et de Radioprotection (ASNR), PSE-SANTE/SESANE/LRTOX, Fontenay aux Roses, F-92260, France; Autorité de Sûreté Nucléaire et de Radioprotection (ASNR), PSE-SANTE/SESANE/LRTOX, Fontenay aux Roses, F-92260, France; Laboratoire des Signaux et Systèmes, Université Paris-Salay, CNRS, Gif-sur-Yvette, 91190, France; Autorité de Sûreté Nucléaire et de Radioprotection (ASNR), PSE-SANTE/SERAMED/LRAcc, Fontenay aux Roses, F-92260, France; Autorité de Sûreté Nucléaire et de Radioprotection (ASNR), PSE-SANTE/SESANE/LRTOX, Fontenay aux Roses, F-92260, France; Autorité de Sûreté Nucléaire et de Radioprotection (ASNR), PSE-SANTE/SESANE/LRTOX, Fontenay aux Roses, F-92260, France; Fondation Jean Dausset–CEPH (Centre d’Etude du Polymorphisme Humain), Paris, 75010, France; Autorité de Sûreté Nucléaire et de Radioprotection (ASNR), PSE-ENV/SERPEN/LECO, Cadarache, Saint-Paul-Lez-Durance, France; Autorité de Sûreté Nucléaire et de Radioprotection (ASNR), PSE-SANTE/SESANE/LRTOX, Fontenay aux Roses, F-92260, France; Autorité de Sûreté Nucléaire et de Radioprotection (ASNR), PSE-SANTE/SERAMED/LRAcc, Fontenay aux Roses, F-92260, France; Autorité de Sûreté Nucléaire et de Radioprotection (ASNR), PSE-SANTE/SESANE/LRTOX, Fontenay aux Roses, F-92260, France; Autorité de Sûreté Nucléaire et de Radioprotection (ASNR), PSE-SANTE/SERAMED/LRAcc, Fontenay aux Roses, F-92260, France

## Abstract

**Purpose:**

In this study, we evaluated 90 bioinformatics pipelines using RNA-Seq datasets from Rats, Zebrafish and Mice. The analysis was conducted in the context of weak signals, including exposure to metallic particles (tungsten), low-dose radiation or medical treatment. RNA-Seq data analysis involves several critical steps, from quality control to differential expression analysis, each offering multiple algorithmic options. Selecting the optimal pipeline is particularly challenging in complex scenarios with weak signals.

We applied a dual strategy based on two complementary approaches to rank and evaluate the performance of these pipelines. The first approach, a widely used method, is based on the correlation between RNA-Seq and qRT-PCR expression data to ensure the direct validation of RNA-Seq results. The second approach leverages machine learning classifiers to rank the pipelines based on their ability to distinguish between exposure groups. This dual strategy was designed to identify the most reliable pipelines capable of providing accurate biological insights, with the top-performing pipeline highlighting key biological processes linked to a weak signal.

**Results:**

Our results highlight the crucial role of pipeline selection in RNA-Seq studies, as it influences both analysis efficiency and biological insights. While our findings are particularly relevant for studies with weak RNA-Seq signals, the ranking methods we employed can be applied in other fields to identify the most appropriate pipeline for generating biologically meaningful data. We provide practical recommendations for bioinformaticians to select robust pipelines, ensuring reliable and insightful outcomes across various research contexts, including environmental exposures.

**Conclusions:**

RNA-Seq pipelines that are effective for strong signals may fail with weak signals or noisy RNA-seq data. Our classifier-based ranking approach is still particularly useful, even when the sequencing depth is lower. Pipelines' sensitivity has a greater impact on counting and normalization than on trimming and mapping. StringTie should be prioritized as a counting method for data with weak signals.

## 1 Introduction

High-throughput sequencing has become increasingly accessible, enabling extensive gene expression analyses across large sets of biological samples at competitive costs (Muir *et al.* 2016), certain research contexts—especially those focusing on subtle biological effects, such as low-dose exposure to pollutants, radiation, or dietary imbalances—require a nuanced approach (Chung *et al.* 2021). In these cases, qRT-PCR (quantitative Reverse Transcription Polymerase Chain Reaction), with its high sensitivity for targeted gene analysis, is often preferred for detecting small but significant expression changes in specific genes (SEQC/MAQC-III Consortium 2014). However, this method is limited by the number of genes it can analyze simultaneously. In contrast, RNA sequencing (RNA-Seq) overcomes these limitations by enabling the sequencing of the entire transcriptome, making it ideal for gene expression profiling analyses that can reveal variation in gene expression between different samples (Muir *et al.* 2016).

Nevertheless, RNA-Seq also has its limitations, especially in studies involving subtle gene expression changes (Rehrauer *et al.* 2013). In such cases, low-coverage reads, though cost-effective, may lack the sensitivity required to detect small variations, making them unsuitable for capturing these weak signals. When high-coverage sequencing is not feasible, optimizing data processing becomes essential to extract relevant signals from biological background noise (Simoneau *et al.* 2021). This optimization enhances the detection of gene expression variation between samples, and helps identify differences between affected and unaffected samples.

Traditionally, RNA-Seq analysis has relied on a variety of bioinformatics tools and pipelines. The rapid development of numerous algorithms for different stages of RNA-Seq analysis—such as read trimming, alignment, read counting, and normalization—has led to a lack of consensus on optimal practices (Conesa *et al.* 2016). Several studies have demonstrated the impact of method choice on RNA-Seq results, underscoring the importance of selecting suitable tools at each step. For example, Nookaew *et al.* (2012) compared various workflows for differential gene expression analysis, while Seyednasrollah *et al.* (2015) evaluated multiple software packages to identify reliable methods. These studies highlight the critical need for careful tool selection to ensure accuracy in results, particularly in complex contexts like low-dose research.

In the face of the complex methodological challenges outlined above, our approach is based on a rigorous strategy to optimize analysis sensitivity based on well-established bioinformatics pipelines. Throughout this work, we refer to a comprehensive study by Corchete *et al.* (2020) that provides a comparative assessment of pipelines for gene expression quantification, offering a detailed ranking based on key metrics derived from genomic data analyses.

On this basis, our study follows a structured approach across several phases. First, we conduct a detailed comparative analysis between the pipeline rankings used on our own data and those outlined in the reference study, applying consistent evaluation criteria for robust comparison.

In the second phase, we extend our analytical framework by integrating additional metrics, allowing a complementary ranking of the pipelines. This step involves the use of advanced classification algorithms, such as Random Forest (RF) and Support Vector Machine (SVM), known for their effectiveness in discriminating complex data patterns. The resulting rankings are then contextualized alongside previous analyses, offering a multifaceted evaluation of the pipelines.

Finally, to assess the biological relevance of these rankings, we perform a functional enrichment analysis. This step evaluates the coherence of our findings with biological significance, ensuring that the highest-ranking pipelines reveal insights consistent with existing literature. Studies like those by Mubeen *et al.* (2022) on gene enrichment analysis have been instrumental in shaping this aspect of our approach. To evaluate the robustness and generalizability of our approach, we reapplied the pipeline comparison on two independent RNA-Seq datasets. The first dataset was obtained based on previous work in our laboratory using Rat models, and the second dataset was obtained from published data using Zebrafish (*Danio rerio*) models.

## 2 Dataset description

### 2.1 Rat model

In our study, the data were obtained from an in vivo experiment conducted on an adult male Sprague-Dawley rat model. Subjects were exposed via inhalation of a particulate aerosol at different doses (low and high) of an environmental chemical pollutant (tungsten) to assess its biological effects on the brain. This project is the subject of an experimental study in our laboratory. The animal ethics committee approved the study protocol, which has been published. More details are available in Macé *et al.* (2024). A dataset comprising 24 subjects, divided into three groups of 8 samples (control, low dose, and high dose), was generated from biopsy extracts by RNA-Seq and qRT-PCR 24 hours post-exposure to tungsten.

### 2.2 Zebrafish model

Murat El Houdigui *et al.* (2019), investigates the molecular effects of chronic ionizing radiation exposure in Zebrafish. RNA-Seq measurements were performed at 24 hours post-fertilisation (hpf) larvae stage and after exposure at three different dose rates of gamma radiation ranging from 0.5 mGy/h to 50mGy/h. We have a total of 26 subjects, divided into four groups (11 controls, 9 exposed to 0.5 mGy/h, 3 exposed to 5 mGy/h, and 3 exposed to 50 mGy/h).

### 2.3 Mice model

Zhao *et al.* (2018), performed RNA-sequencing on the prefrontal cortex of MK-801-exposed male Mice in order to analyze gene expression and co-expression patterns related to schizophrenia and to identify mechanisms that underlie the molecular etiology of this disorder. Prefrontal cortex mRNA profiles of three saline control Mice and six MK801-exposed Mice were generated by RNA-seq, using Illumina X-10.

Further details about these data can be found at Supplementary Material Section 1.

## 3 Methods

### 3.1 RNA-Seq data analysis

**Figure 1:**
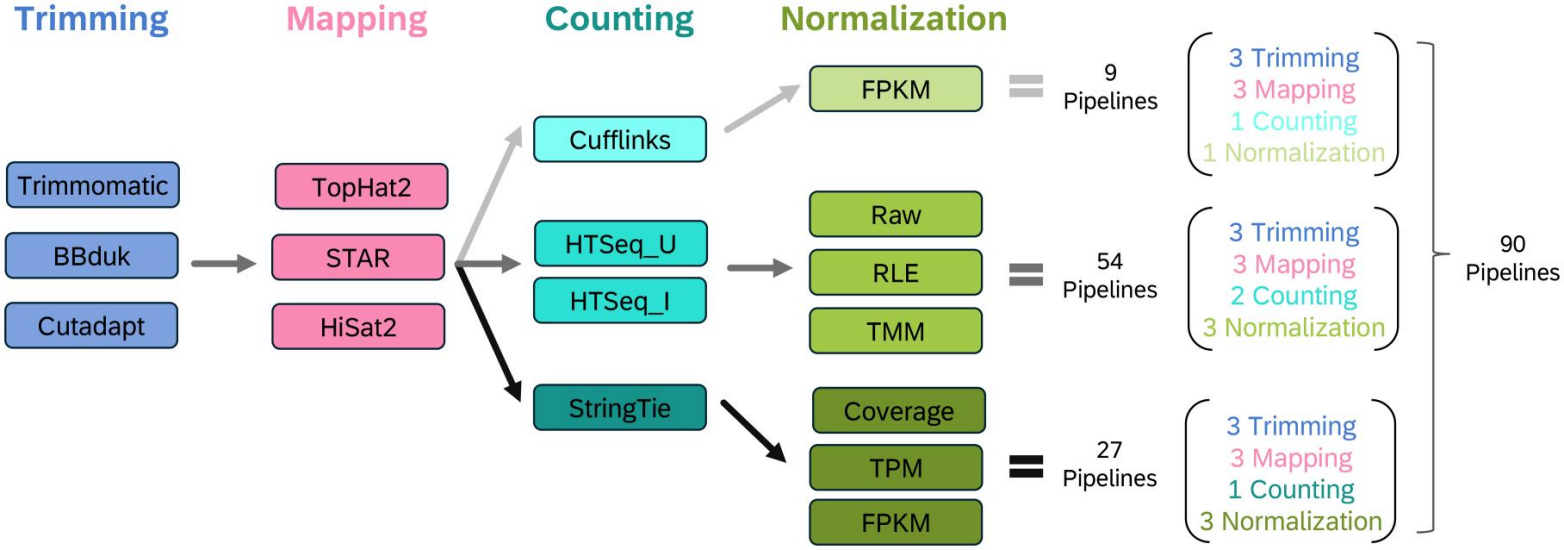
RNA-Seq analysis workflows: combinatorial exploration of algorithms and methods for trimming, mapping, counting, and normalization. Hues of one color indicate groups of algorithms for each step, and arrows indicate possible combinations of methods across steps. In particular, not all normalization methods are compatible with all counting algorithms. Each arrow represents a multiplied combination, resulting in a total of 90 pipelines.

Our work is distinguished by a comprehensive evaluation of 90 pipelines, derived from a carefully curated combination of three trimming methods, three aligners, four counting techniques, and six normalization approaches as illustrated in Fig. 1. These pipelines are genome alignment-based and correspond to those presented in the reference study by Corchete *et al.* (2020), enabling a robust comparative analysis. Each component was meticulously selected based on its validation and prominence within the scientific community, as reflected in its citation frequency in recent literature. Additionally, the relevance and effectiveness of these methods are supported by studies such as Jiang *et al.* (2024), which systematically evaluates various combinations of trimming, mapping, and counting tools, and Liu *et al.* (2022), which highlights the importance of tool selection across multiple RNA-Seq datasets. Details about our pipeline combinations can be found in Supplementary Material Sections 2 and 5.

### 3.2 Pipeline assessment based on precision and accuracy analysis

In our context, which focuses on low-dose exposure data, it is essential to evaluate whether the top-performing pipelines for oncological datasets are also optimal for our data, which pertain to a different thematic area. This approach allows us to assess if the ranking of Corchete *et al.* (2020) needs to be adapted to our specific case. Moreover, recent studies, such as that by Alonso-Peña *et al.* (2022), have cited Corchete *et al.*’s work as a reference and have used their top-ranked algorithms to select specific methods for different steps of the analysis pipeline, highlighting the influence of this benchmark across various research domains. In their benchmark study, Corchete *et al.* (2020) conducted a comprehensive comparative assessment of pipelines for gene expression quantification, providing a detailed ranking based on key metrics derived from oncological data analyses. For our first ranking metric, we used the two metrics directly drawn from this work based on *precision* and *accuracy* analyses. Briefly, a lower rank precision index indicates better pipeline stability, meaning lower variability of obtained results while a lower accuracy rank index indicates better pipeline consistency with the reference method qRT-PCR.

**Figure 2:**
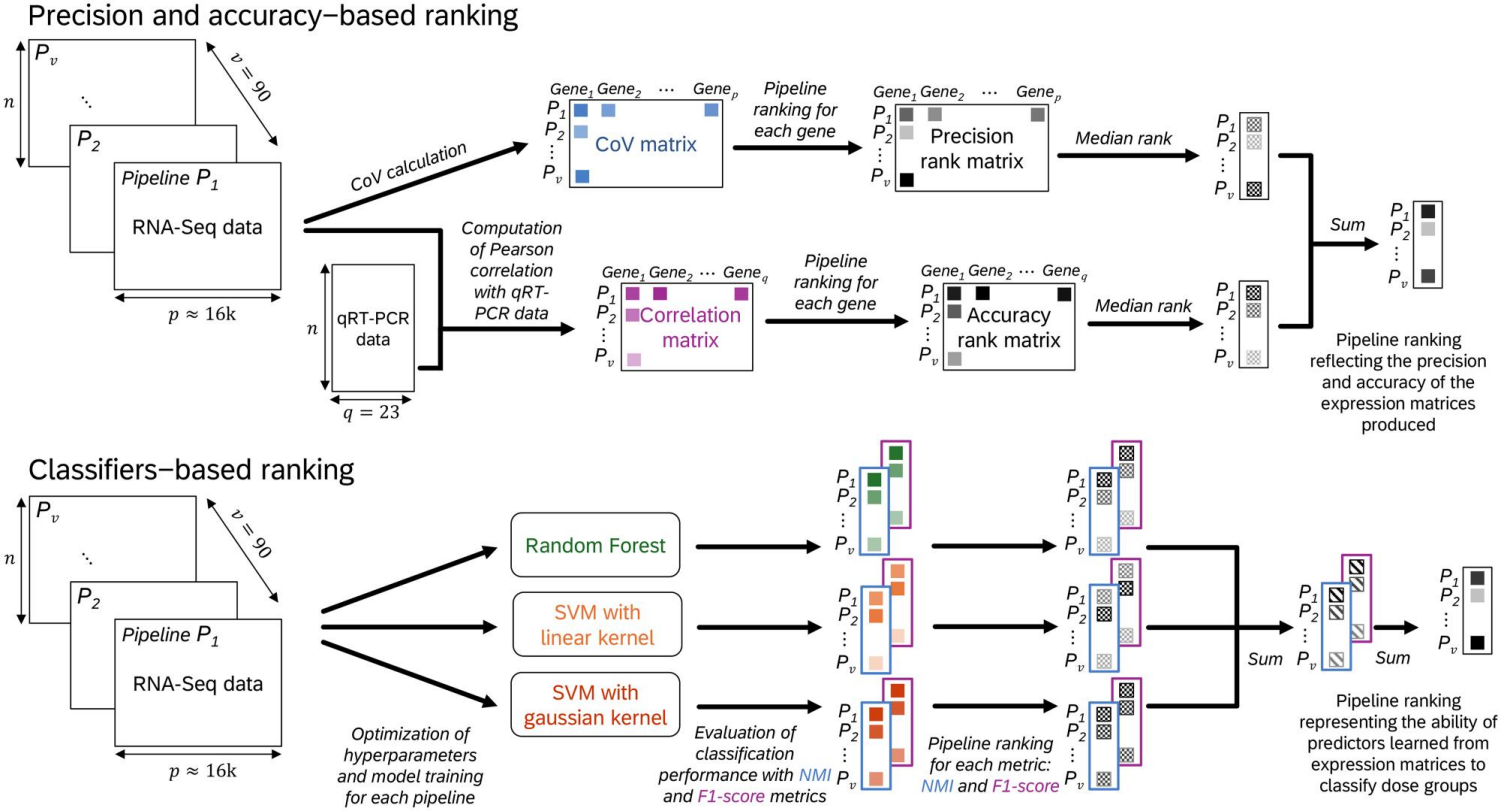
Benchmark procedure for the evaluation of RNA-Seq pipelines: (top) Precision and accuracy-based ranking, (bottom) Classifiers-based ranking (SVM = Support Vector Machine, NMI = Normalized Mutual Information).

The best pipelines were determined by considering the sum of rankings from both precision and accuracy metrics. Each value was weighted equally, ensuring that the selected pipelines excel in both metrics. The lower this sum, the better the pipeline’s performance. This Precision-Accuracy procedure is graphically illustrated in the top pannels of Fig. 2. Details can be found in Corchete *et al.* study (Corchete *et al.* 2020) and Supplementary Material Section 3.

### 3.3 Pipeline assessment based on classification performance

In this section, we propose a new ranking metric based on classifiers to evaluate the 90 bioinformatics pipelines. Each pipeline generated a data matrix, where columns represent genes (features), and rows represent samples from distinct experimental conditions. The goal was to determine how well each pipeline could correctly classify samples according to these conditions. The strategies chosen for classification are described in details in Supplementary Material Section 4. To summarize, Random forests (RF) and Support Vector Machines (SVM) classifiers were used to discriminate the experimental conditions of each dataset (Rat, Zebrafish and Mice) using each pipeline’s data matrix. Three metrics were used to assess the performance of these classifications: the Normalized Mutual Information (NMI), the F1-Score and the the effect size of differential gene expression (DGE). The classifier ranking was firstly established separatly for NMI and the F1-score for each classifier by assigning rank 1 to the one with the highest metric value. The sum of NMI and F1-Score rank metrics was then used as fair weighting of classifier performance. Where the merging of rankings resulted in ties, an additional ranking step was added based on the DGE to break the ties. We only considered the ranking based on the number of DGE when two or more pipelines had the same rank. Indeed, using the number of DGE as as a principal ranking criterion has certain limitations especially after p-value correction: the number of significant genes is sometimes insufficient for weak signals, or even zero. However, incorporating the DGE criterion makes possible to distinguish between pipelines that are ranked as tied by classifiers (see Table 4). This rigorous and balanced evaluation strategy ensure that the selected pipelines are distinguished by their stability across the different classifiers.

**Table 1:** Top ranked pipelines based on precision and accuracy metrics, applied to the Macé *et al.* (2024) dataset.

Corchete *et al.* ranking	Trimming	Mapping	Counting	Normalization	Precision	Accuracy	Sum	Rank
**96 (53)**	Bbduk	TopHat2	StringTie	TPM	15,5	25	40,5	1
**185 (85)**	Cutadapt	TopHat2	StringTie	Coverage	23	19	42	2
**101 (56)**	Cutadapt	TopHat2	StringTie	TPM	16,5	26	42,5	3
**103 (58)**	Cutadapt	TopHat2	StringTie	FPKM	10,5	33	43,5	4
**135 (67)**	Trimmomatic	TopHat2	StringTie	Coverage	25,5	19	44,5	5
**140 (68)**	Bbduk	TopHat2	StringTie	Coverage	26	19	45	6
**105 (59)**	Bbduk	TopHat2	StringTie	FPKM	12	33	45	7
**99 (55)**	Trimmomatic	TopHat2	StringTie	FPKM	14	33	47	8
**150 (71)**	Bbduk	STAR	StringTie	Coverage	20	28	48	9
**60 (39)**	Cutadapt	STAR	StringTie	TPM	15	33	48	10

The ranking extracted from Corchete *et al.* (2020) is presented both in relation to the 192 pipelines tested in said study and, in parenthesis, in relation to the 90 pipelines tested in the present study.

**Table 2:** Top-ranked pipelines based on F1 classification performance and NMI similarity metric, applied to the Macé *et al.* (2024) dataset.

Corchete *et al.* ranking	Trimming	Mapping	Counting	Normalization	Rank F1	Rank NMI	Sum	Rank DGE	Rank
**135 (67)**	Trimmomatic	TopHat2	StringTie	Coverage	7	1	8	63	1
**26 (20)**	BBduk	STAR	StringTie	FPKM	12	4	16	75	2
**105 (59)**	BBduk	TopHat2	StringTie	FPKM	8	8	16	77	3
**185 (85)**	Cutadapt	TopHat2	StringTie	Coverage	1	15	16	81	4
**103 (58)**	Cutadapt	TopHat2	StringTie	FPKM	5	12	17	38	5
**20 (14)**	BBduck	STAR	StringTie	TPM	4	14	18	33	6
**153 (74)**	Trimmomatic	STAR	StringTie	Coverage	13	5	18	33	7
**150 (71)**	BBduck	STAR	StringTie	Coverage	17	9	26	45	8
**186 (86)**	Cutadapt	STAR	StringTie	Coverage	20	6	26	60	9
**111 (64)**	Cutadapt	HiSat2	StringTie	FPKM	6	21	27	53	10

RF = Random Forests, SVM.L = Support Vector Machine with Linear kernel, SVM.G = Support Vector Machine with Gaussian kernel. The ranking reported by Corchete *et al.* (2020) is shown both relative to the 192 pipelines evaluated in their study and, in parentheses, relative to the 90 pipelines assessed in the present analysis.

**Table 3:** Top-ranked pipelines based on F1 classification performance and NMI similarity metric, applied to the independent Murat El Houdigui *et al.* (2019) dataset.

Corchete *et al.* ranking	Trimming	Mapping	Counting	Normalization	Rank F1	Rank NMI	Sum	Rank DGE	Rank
**106 (61)**	BBduk	HiSat2	StringTie	FPKM	1	1	2	25	1
**112 (65)**	Trimmomatic	HiSat2	StringTie	TPM	2	2	4	58	2
**25 (19)**	Trimmomatic	STAR	StringTie	FPKM	3	3	6	72	3
**35 (26)**	Trimmomatic	STAR	StringTie	TPM	4	4	8	86	4
**13 (11)**	Trimmomatic	TopHat2	HTSeq_U	TMM	5	5	10	31	5
**98 (54)**	Trimmomatic	TopHat2	StringTie	TPM	6	6	12	6	6
**107 (62)**	Trimmomatic	HiSat2	StringTie	FPKM	11	7	18	50	7
**66 (43)**	Trimmomatic	TopHat2	HTSeq_IN	RLE	12	8	20	16	8
**99 (55)**	Trimmomatic	TopHat2	StringTie	FPKM	13	9	22	71	9
**11(10)**	Trimmomatic	HiSat2	HTSeq_U	TMM	19	12	31	30	10

RF = Random Forests, SVM.L = Support Vector Machine with Linear kernel, SVM.G = Support Vector Machine with Gaussian kernel. For comparison, the ranking reported by Corchete *et al.* (2020) is shown both relative to the 192 pipelines evaluated in their study and, in parentheses, relative to the 90 pipelines assessed in the present analysis.

**Table 4:** Top-ranked pipelines based on F1 classification performance and NMI similarity metric, applied to the independent Zhao *et al.* (2018) dataset.

Corchete *et al.* ranking	Trimming	Mapping	Counting	Normalization	Rank F1	Rank NMI	Sum	Rank DGE	Rank
**186 (86)**	Cutadapt	STAR	StringTie	Coverage	1	1	2	10	1
**82 (49)**	BBduk	TopHat2	Cufflinks	FPKM	2	2	4	4	2
**37 (25)**	BBduk	HiSat2	HTSeq_U	RLE	2	2	4	6	3
**13 (11)**	Trimmomatic	TopHat2	HTSeq_U	TMM	2	2	4	8	4
**40 (29)**	Cutadapt	STAR	HTSeq_IN	RLE	2	2	4	12	5
**39 (28)**	Cutadapt	STAR	HTSeq_IN	TMM	2	2	4	12	6
**103 (58)**	Cutadapt	TopHat2	StringTie	FPKM	3	3	6	8	7
**105 (59)**	BBduk	TopHat2	StringTie	FPKM	3	3	6	11	8
**66 (43)**	Trimmomatic	TopHat2	HTSeq_IN	RLE	3	3	6	13	9
**150 (71)**	BBduk	STAR	StringTie	Coverage	3	5	8	9	10

RF = Random Forests, SVM.L = Support Vector Machine with Linear kernel, SVM.G = Support Vector Machine with Gaussian kernel. For comparison, the ranking reported by Corchete *et al.* (2020) is shown both relative to the 192 pipelines evaluated in their study and, in parentheses, relative to the 90 pipelines assessed in the present analysis.

## 4 Results

### 4.1 Dual approach to evaluating the performance of the transcriptomic dataset from rats model

#### 4.1.1 Precision and accuracy for the evaluated pipelines

Table 1 shows the top 10 bioinformatics pipelines identified from our RNA-Seq and qRT-PCR data analysis. The performance of the RNA-Seq pipelines was assessed using accuracy and precision criteria on 23 genes (*P *= 23) evaluated across eight control samples from the frontal cortex (FC) (*n *= 8), see Table S5. This ranking was based on two key metrics: accuracy and precision. It is important to recall that among the 90 bioinformatics pipelines tested, we evaluated four different counting algorithms (see Fig. 1). Notably, StringTie was consistently present in the top 10 identified pipelines. This means StringTie demonstrated superior efficiency in these configurations, regardless of the other pipeline parameters. This outcome was not pre-conceived but emerged naturally from our rigorous testing, indicating that StringTie plays a key role in achieving high performance according to both precision and accuracy metrics evaluated in this study.

#### 4.1.2 Classification performance for the evaluated pipelines

**Figure 3:**
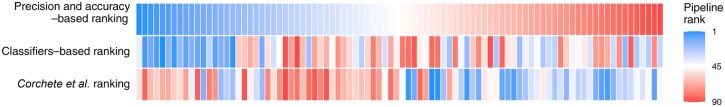
Comparison of bioinformatic pipeline performances on the rat transcriptomic datasets across three ranking strategies: (i) a classifier-based ranking combining NMI and F1 score metrics, (ii) a precision and accuracy–based ranking, and (iii) the ranking reported by Corchete *et al*. (2020). Ranks are represented as a heatmap (blue = high-performing pipelines). Pipeline names are omitted on the x-axis to prevent visual clutter.

Table 2 presents the ranking of the top 10 bioinformatics pipelines based on classification accuracy, measured using the NMI and F1 score metrics. More details can be found in Supplementary Material Section 6. Note firstly that the StringTie counting method is the common element among all the top 10 pipelines in this analysis. Once again, this highlights the effectiveness of StringTie as a key component for achieving high performance, whether in terms of correlation with qRT-PCR data or classification performance. To better understand and compare the results of the 90 pipelines tested, it is essential to visualize the rankings as a whole. With this in mind, we developed the heatmap shown in Fig. 3. This graphic aims to unify the results of the two rankings we performed with those from the reference article. The visual analysis of the heatmap reveals a striking contrast: the top-ranked pipelines in the reference article often rank among the lowest in our ranking, while those we identified as the best ones rank among the worst in the reference article (Corchete *et al.* 2020). It is also worth noting that the two ranking methods we used—one based on correlation and variability metrics, and the other on classification performance—show relatively consistent results. A key observation is the near-complete opposition in rankings between the two studies. This striking contrast underscores the impact of dataset-specific factors, highlighting the need to adapt pipeline evaluations to the context of the study. These differences emphasize the specific challenges associated with radiation protection data compared to other fields, such as oncology.

### 4.2 Validation of pipeline robustness

Firstly, we investigated the biological pathways enriched in the expression matrix produced by each pipeline to determine which ranking is relevant in our context. This will be discussed in detail in the next section, where we will evaluate not only the pipelines’ performance from a statistical perspective but also their ability to produce biologically meaningful and reliable results. This step is crucial for deciding between the different ranking approaches and identifying the pipelines that offer optimal statistical performance and biological relevance within our study. For the second trial, our aim was to reproduce Stringie’s impact using a comparable database where the signal was weaker, but with greater depth. This decision was motivated by our earlier finding that Stringie was the most relevant counting method in both classification approaches, as well as by published work emphasising its reliability in cases of a weak signal (Liu *et al.* 2022).

#### 4.2.1 Functional enrichment evaluation

##### 4.2.1.1 Multivariate statistical analysis

**Figure 4:**
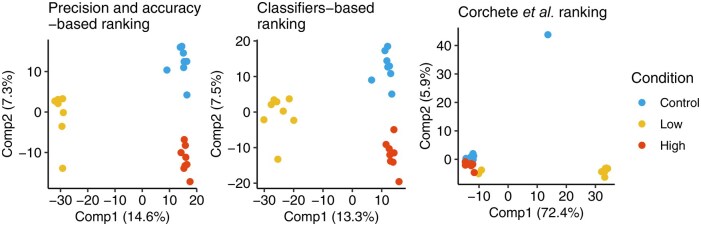
First factorial planes of sPLS-DA performed on the datasets obtained with the top-ranked pipelines according to each of the three different ranking methods. sPLS-DA shows the discrimination of samples based on experimental conditions (Control, High, Low), with the variance explained by the first two latent components indicated on the axes.

To evaluate the relevance of the pipelines in separating biological classes, we retained the top-ranked pipelines of the three different approaches: the ranking based on precision and accuracy metrics, the ranking based on supervised classifier performance and the ranking from the reference paper (Corchete *et al.* 2020). To visualize each pipeline’s ability to discriminate between the different conditions (Control, High, Low), we performed a Sparse Partial Least Squares Discriminant Analysis (sPLS-DA) for each pipeline’s count data matrix. sPLS-DA was used initially to observe the global group separation achieved by each pipeline and to better understand the specific genes that contribute the most to the separation of biological conditions. The sparsity-driven approach in sPLS-DA focuses on a subset of relevant genes while reducing noise and the inherent complexity of high-dimensional RNA-Seq data. The application of sparsity requires that most gene coefficients are set to zero, leaving only the most influential genes for constructing discriminant models, simplifying the interpretation and focusing the analysis on the most significant genes (Lê Cao *et al.* 2011). We implemented sPLS-DA using the mixOmics package in R (Rohart *et al.* 2017). The model was constructed by selecting a fixed number of genes per component, thereby defining a subset of genes that maximizes separation between conditions. The model was evaluated using repeated cross-validation (1000 repetitions with 5-fold cross-validation), allowing us to assess the model’s performance and verify the stability of the selected genes. The most stable genes, those consistently retained across cross-validation iterations, were selected for the final modeling. Figure 4 illustrates the results of the sPLS-DA, highlighting notable differences in each pipeline’s ability to separate biological classes. As expected, the best pipeline, according to the classifier performance, shows a clear separation of the three conditions, with strong discrimination on the two main axes. The best-ranking pipeline according to the precision and accuracy metrics also shows good separation, though with slightly different explained variances. Finally, the pipeline ranked highest according to the reference article presents a less clear separation of classes and a possible presence of outliers, suggesting limited suitability.

##### 4.2.1.2 Functional enrichment analysis

**Figure 5:**
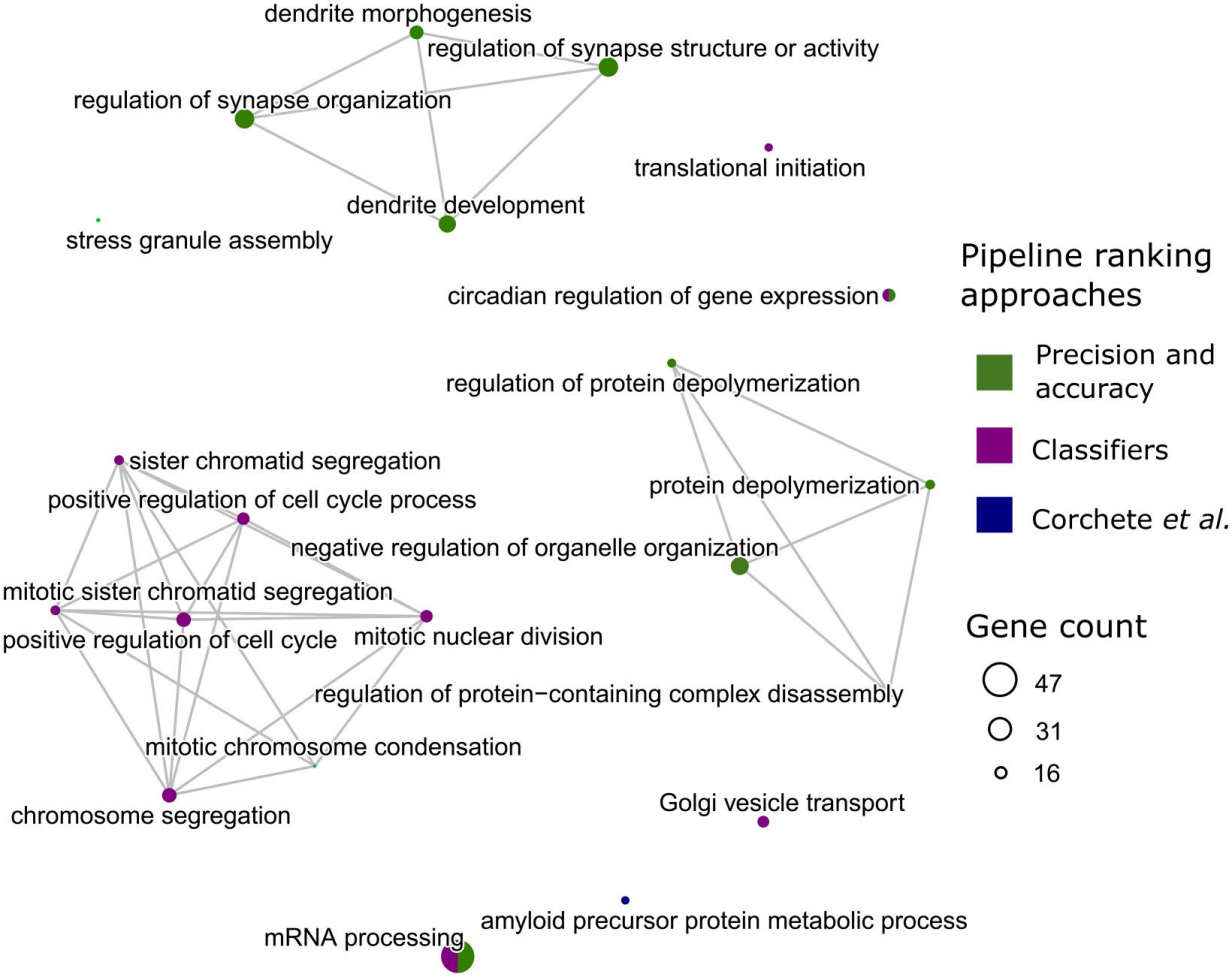
Enriched Gene Ontology-terms network computed on the stable genes identified in Comp1 from sPLS-DA analyses. Nodes represent GO terms for biological pathways, and the thickness of the segments between them indicates the sharing of genes.

In this section, we investigate whether the multivariate analyses conducted above were able to provide insights into each pipeline’s ability to discriminate biological classes. Indeed, functional enrichment analysis allows us to discuss and interpret the results with biologists from our team. The enrichment analysis was conducted using the enrichGO function, which utilizes Entrez gene identifiers sourced from the org. Rn.eg.db database, an R package providing up-to-date annotations for Rat genes (*Rattus norvegicus*). We thus ensure that our enrichment analysis incorporates the latest available data for this model species. In Fig. 5, we visualize enriched biological processes for stable genes identified in the first principal component (PC1) of the sPLS-DA analysis. This component was of interest since it differentiated the “Low exposure” group of animals from the others. While several GO terms were common across pipelines, further analysis reveals that the classifier-based pipeline captures additional processes crucial for distinguishing the studied conditions. More precisely, the analysis conducted on data from the top-ranked pipeline for classification revealed that the exposure primarily impacted neuromorphological, inflammatory, cellular, and nuclear processes. These findings were verified in consultation with biologists, who confirmed the interest of these results, noting consistent observations in experiments conducted at the cell level in the laboratory. For instance, chromosome condensation and segregation, as well as dendrite and synapse organization are active processes in neuronal cell homeostasis. These results are particularly relevant, as they suggest that these processes may be key mechanisms in the observed biological response of neuronal cells to tungsten toxicity (Macé *et al.* 2024). The processes revealed by the classifiers method are of particular interest, as chromatin remodeling has been observed in a phenotype of suffering cortical neurons. Thus, the best-ranked classification pipeline provided a comprehensive and relevant perspective, allowing a better understanding of the cellular effects of chemical particle exposure and supporting the establishment of a robust molecular signature for classifying exposure groups.

#### 4.2.2 Validation of pipeline robustness on independent transcriptomic datasets

According to our results from the first dataset, the rankings obtained from qRT-PCR and from the classifier-based approach were largely consistent. We also found that the pipeline ranked highest by the classifier approach was biologically more informative. For this reason, we evaluated reproducibility on independent datasets, primarily to validate the robustness of the classifier-based ranking strategy. To evaluate the generalizability of our ranking framework, we re-applied the pipeline comparison to independent RNA-Seq datasets published in the literature, where Murat El Houdigui *et al.* (2019) investigate the molecular effects of chronic ionizing radiation exposure in Zebrafish and Zhao *et al.* (2018) examined the effects of MK-801 treatment on gene expression in prefrontal cortex (PFC) of Mice. These databases contain only RNA-seq data reflecting the fact that, in biological research, obtaining both qRT-PCR and RNA-Seq measurements from the same sample is often difficult, especially across different laboratories, which further supports the need for alternative validation approaches. Consequently, the pipeline ranking based on precision and accuracy cannot be computed (since it requires additionnal qRT-PCR data). In contrast, the classifier’s approach can be applied using only RNA-Seq and detailed top-10 ranked pipelines obtained using this approach is presented below in Tables 3 and 4 and Supplementary Material Section 6.

**Figure 6:**
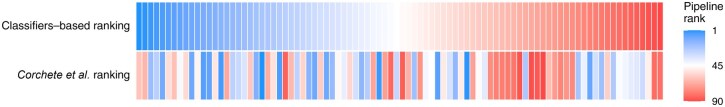
Comparison of bioinformatic pipeline performances on the Zebrafish (*Danio rerio*) transcriptomic datasets across two ranking strategies: (i) a classifier-based ranking combining NMI and F1 score metrics and (ii) the ranking reported by Corchete *et al*. (2020). Ranks are represented as a heatmap (blue = high-performing pipelines). Pipeline names are omitted on the x-axis to prevent visual clutter.

The stability of StringTie and FPKM based configurations across both performance metrics and datasets highlights their reliability for transcript quantification and normalization in RNA-Seq analysis pipelines. Overall, these validations on an independent Murat El Houdigui *et al.* (2019) dataset confirm the reproducibility and transferability of the proposed ranking framework as illustrated in Fig. 6.

**Figure 7:**
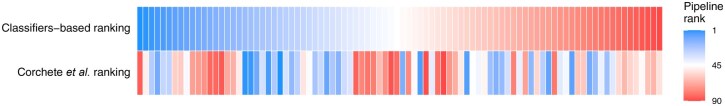
Comparison of bioinformatic pipeline performances on Mice transcriptomic datasets across two ranking strategies: (i) a classifier-based ranking combining NMI and F1 score metrics and (ii) the ranking reported by Corchete *et al*. (2020). Ranks are represented as a heatmap (blue = high-performing pipelines). Pipeline names are omitted on the x-axis to prevent visual clutter.

Similarly, the Zhao *et al.* (2018) dataset confirms stability with respect to StringTie, aligning with previous work demonstrating its sensitivity to subtle transcriptional changes (Liu *et al.* 2022). Conclusions about normalization are less straightforward because many pipelines have the same classification rank, see Supplementary Material Section 6. This is probably due to the weak signal and, more importantly, the imbalanced design with few samples. Finally, we noticed that the data depth is much lower than in the other two databases. This last dataset study highlights again that the choice of bioinformatics pipeline must be adapted to the specific properties of each dataset, as illustrated in Fig. 7.

## 5 Discussion

This study highlights the significant impact of pipeline selection on RNA-Seq data analysis, particularly in contexts characterized by weak transcriptional signals, as in this study for low-dose metallic particle exposure and chronic ionizing radiation effects. By evaluating 90 bioinformatics pipelines using two complementary approaches—correlation with qRT-PCR data and classification performance—we were able to identify optimal configurations that ensure both analysis robustness and biological relevant observation. This finding was confirmed on independent datasets investigating a similar weak signal context.

One of the most consistent findings across our analyses is the superior performance of pipelines that incorporate StringTie for the counting step. StringTie’s ability to accurately assemble transcripts and quantify isoforms proved essential to detecting tenuous expressed genes, which are often difficult to capture in datasets with weak signal fluctuations. This finding underscores the biological relevance of StringTie in environmental exposure studies. StringTie consistently appeared in the top-performing pipelines regardless of the trimming, mapping, or normalization methods used, further reinforcing its utility in RNA-Seq workflows.

Normalization methods also played a critical role in pipeline performance. While TMM normalization has been widely recommended in oncological studies for addressing library composition biases (Robinson and Oshlack 2010), our results revealed that FPKM was better suited to low-dose exposure data as ranks as the most common pipeline within the top 10 across both Rat and Zebrafrish databases.

Sequencing depth emerged as another key factor influencing the performance of RNA-Seq pipelines. In datasets characterized by weak signals, insufficient sequencing depth can hinder the detection of biologically meaningful changes, even with optimal pipeline configurations (Baccarella *et al.* 2018). Based on our results from the three datasets, we observed that in the Zebrafish dataset—where the sequencing depth is high—the pipelines appear less sensitive to the pipeline combinations in term of classification accuracy. We also noted that NMI and F1-Score metrics produce almost identical rankings in this case (see Table 3).

Our classifier-based ranking approach still particularly useful, even when the sequencing depth is lower, since it can extract meaningful information. Indeed, the study of the Rat database which combines low sequencing depth (20 million reads per sample—chosen primarily for cost-related reasons) and weak signal strength successfully revealed significant biological insights. The results, validated by other cellular analyses (Macé *et al.* 2024) demonstrate that even low-depth sequencing data can yield meaningful and reliable findings when analyzed with tailored pipelines. Furthermore, the proposed classifier-based ranking overcomes the limitations of the precision-accuracy ranking approach which needs a qRT-PCR gold standard database with two major constraints: financial contraints and identified genes which may not overlap with those detected via RNA-Seq.

However, when weak signal is combined with few imbalanced data, the classification reaches its limits and the DGE-based criterion provides additional and complementary information for classifying the pipelines as illustrated in the Mice study.

Our findings offer practical guidance for bioinformaticians and researchers working in similar contexts. First, StringTie should be prioritized as a counting method due to its consistent performance across both correlation and classification-based evaluations. Second, normalization strategies represent a critical aspect to consider, as their choice significantly impacts the robustness of the results; FPKM-based method has shown particular effectiveness in low-dose exposure studies. Finally, pipeline configurations optimized for other research areas should not be directly applied without validation in the specific context of interest. Instead, careful evaluation and adaptation are required to ensure robust and reliable results.

## 6 Conclusion

Facing the complex challenges posed by analyzing the biological effects of environmental low-dose exposure to pollutants, this work underscores the importance of adapting the analytical strategy to the physical constraints intrinsically linked to studies, such as those concerned with the toxicology of environmental exposures. Our findings open several avenues for perspectives in the optimization of highly noisy data, whether they result from insufficient analytical resolution (lack of reading depth), biological characteristics with low levels of fluctuation, or both. First, future work should need to test these pipelines on other datasets to validate this proof of concept across different contexts. Second, exploring alternative bioinformatics approaches, such as transcriptome-focused analyses, pipelines combining genome and transcriptome data, or pseudo-alignment methods, could further enhance the adaptability and precision of pipeline configurations tailored to weak signal detection.

## Supplementary Material

vbag134_Supplementary_Data

## Data Availability

FASTQ Rat dataset and the normalized count matrix obtained by the best pipeline are available at GSE313664. FASTQ and metadata files of the Zebrafish (*Danio rerio*) and Mice RNA-Seq datasets are available at GSE134634 and GSE111708. Code for most routines is available on GitHub: https://github.com/Malekbaroudi/RADPIPEX.

## References

[vbag134-B1] Alonso-Peña M , Espinosa-Escudero R, Hermanns HM et al Impact of liver inflammation on bile acid side chain shortening and amidation. Cells 2022;11:3983. https://www.mdpi.com/2073-4409/11/24/3983. 36552746 10.3390/cells11243983 PMC9777420

[vbag134-B2] Baccarella A , Williams CR, Parrish JZ et al Empirical assessment of the impact of sample number and read depth on RNA-Seq analysis workflow performance. BMC Bioinformatics 2018;19:423. 10.1186/s12859-018-2445-2 30428853 PMC6234607

[vbag134-B3] Chung M , Bruno VM, Rasko DA et al Best practices on the differential expression analysis of multi-species RNA-Seq. Genome Biol 2021;22:121. 10.1186/s13059-021-02337-8 33926528 PMC8082843

[vbag134-B4] Conesa A , Madrigal P, Tarazona S et al A survey of best practices for RNA-Seq data analysis. Genome Biol 2016;17:13–9. 10.1186/s13059-016-0881-8 26813401 PMC4728800

[vbag134-B5] Corchete LA , Rojas EA, Alonso-López D et al Systematic comparison and assessment of RNA-Seq procedures for gene expression quantitative analysis. Sci Rep 2020;10:19737. 10.1038/s41598-020-76881-x 33184454 PMC7665074

[vbag134-B6] Jiang G , Zheng J-Y, Ren S-N et al A comprehensive workflow for optimizing RNA-Seq data analysis. BMC Genomics 2024;25:631. 10.1186/s12864-024-10414-y 38914930 PMC11197194

[vbag134-B7] Lê Cao K-A , Boitard S, Besse P. Sparse PLS discriminant analysis: biologically relevant feature selection and graphical displays for multiclass problems. BMC Bioinformatics 2011;12:253–17. 10.1186/1471-2105-12-253 21693065 PMC3133555

[vbag134-B8] Liu X , Zhao J, Xue L et al A comparison of transcriptome analysis methods with reference genome. BMC Genomics 2022;23:232. 10.1186/s12864-022-08465-0 35337265 PMC8957167

[vbag134-B9] Macé L , Brizais C, Bachelot F et al Exposure to tungsten particles via inhalation triggers early toxicity marker expression in the rat brain. Inhal Toxicol 2024;36:261–74. 10.1080/08958378.2024.2349895 38836331

[vbag134-B10] Mubeen S , Tom Kodamullil A, Hofmann-Apitius M et al On the influence of several factors on pathway enrichment analysis. Brief Bioinform 2022;23:bbac143. 10.1093/bib/bbac143 35453140 PMC9116215

[vbag134-B11] Muir P , Li S, Lou S et al The real cost of sequencing: scaling computation to keep pace with data generation. Genome Biol 2016;17:53–9. 10.1186/s13059-016-0917-0 27009100 PMC4806511

[vbag134-B12] Murat El Houdigui S , Adam-Guillermin C, Loro G et al A systems biology approach reveals neuronal and muscle developmental defects after chronic exposure to ionising radiation in zebrafish—dataset used as input for the present analysis. Sci Rep 2019;9:20241. 31882844 10.1038/s41598-019-56590-w PMC6934629

[vbag134-B13] Nookaew I , Papini M, Pornputtapong N et al A comprehensive comparison of RNA-Seq-based transcriptome analysis from reads to differential gene expression and cross-comparison with microarrays: a case study in *Saccharomyces cerevisiae*. Nucleic Acids Res 2012;40:10084–97. 10.1093/nar/gks804 22965124 PMC3488244

[vbag134-B14] Rehrauer H , Opitz L, Tan G et al Blind spots of quantitative RNA-Seq: the limits for assessing abundance, differential expression, and isoform switching. BMC Bioinformatics 2013;14:370–10. 10.1186/1471-2105-14-370 24365034 PMC3879183

[vbag134-B15] Robinson MD , Oshlack A. A scaling normalization method for differential expression analysis of RNA-Seq data. Genome Biol 2010;11:R25–9. 10.1186/gb-2010-11-3-r25 20196867 PMC2864565

[vbag134-B16] Rohart F , Gautier B, Singh AS et al mixOmics: an R package for ’omics feature selection and multiple data integration. PLoS Comput Biol 2017;13:e1005752. https://journals.plos.org/ploscompbiol/article?id=10.1371/journal.pcbi.1005752 29099853 10.1371/journal.pcbi.1005752 PMC5687754

[vbag134-B17] SEQC/MAQC-III Consortium. A comprehensive assessment of RNA-Seq accuracy, reproducibility and information content by the sequencing quality control consortium. Nat Biotechnol 2014;32:903–14. 25150838 10.1038/nbt.2957 PMC4321899

[vbag134-B18] Seyednasrollah F , Laiho A, Elo LL. Comparison of software packages for detecting differential expression in RNA-Seq studies. Brief Bioinform 2015;16:59–70. 10.1093/bib/bbt086 24300110 PMC4293378

[vbag134-B19] Simoneau J , Dumontier S, Gosselin R et al Current RNA-Seq methodology reporting limits reproducibility. Brief Bioinform 2021;22:140–5. 10.1093/bib/bbz124 31813948 PMC7820846

[vbag134-B20] Zhao J , Liu X, Huo C et al Abnormalities in prefrontal cortical gene expression profiles relevant to schizophrenia in MK-801-exposed C57BL/6 mice. Neuroscience 2018;390:60–78. 10.1016/j.neuroscience.2018.07.046. https://www.sciencedirect.com/science/article/pii/S0306452218305219. 30102956

