# Supplementary material for: A dual approach to evaluate the performance of RNA-Seq data analysis pipelines with weak signals
Source: Bioinform Adv. 2026 May 9;6(1):vbag134. doi: 10.1093/bioadv/vbag134 (PMC13242180; doi:10.1093/bioadv/vbag134)
Supplement: vbag134_Supplementary_Data [file vbag134_supplementary_data.pdf]

This Supplementary Material accompanies the manuscript *"A Dual Approach to Evaluate the Performance of RNA-Seq Data Analysis Pipelines with Weak Signals"*

1 Transcriptomic and qRT-PCR data in the preclinical Models

1.1 Rat Model

1.1.1 Transcriptomic Data

Total RNA was extracted from frontal cortex tissue using an RNAEasy Kit (Qiagen), and isolation of polyadenylated RNA (mRNA) from total RNA samples was performed with the same kit. RNA-Sequence libraries were prepared with the TruSeq Stranded mRNA Prep Kit (Illumina, San Diego, CA, USA). Sequencing was performed at Genewiz Azenta Life Sciences (Leipzig, Germany) on a NovaSeq 6000 machine (Illumina) to generate at least 20 million reads. In this study, the sequencing coverage was limited to 20,000,000 reads per sample due to budgetary constraints, a compromise that, although cost-effective, posed challenges in achieving sufficient data coverage and accurate detection of low expression levels.

1.1.2 qRT-PCR Data

To assess differential gene expression using Real-Time Quantitative Reverse Transcription Polymerase Chain Reaction (qRT-PCR), biologists selected 23 genes based on their specific biological function and its relevance to low-dose exposure and neurotoxicity processes. The qRT-PCR is a sensitive and precise method used to quantify gene expression by measuring the mRNA levels of target genes. The RNA extracted from the samples is first converted into complementary DNA (cDNA) using reverse transcriptase, then amplified by real-time PCR. This technique provides cycle threshold (Ct) values, which reflect the initial amount of mRNA, and is used here to compare the expression levels of the selected genes. The threshold cycle or Ct of the genes of interest and reference genes (here Gapdh, Hprt and Beta-actin) was used to process the results. Relative expression was calculated using the ΔCt method by normalizing with respect to the three housekeeping genes as presented in (Macé et al. 2024).

1.2 Zebrafish model

FASTQ and metadata files from an RNA-Seq dataset from samples of zebrafish (*Danio rerio*) were obtained from GSE134634. After downloading and decompressing the datasets, the integrity of the data was checked and quality control was performed with FastQC. In this study, the sequencing coverage was 40,000,000 reads per sample, indicating high data quality.

1.3 Mice model

An RNA-seq dataset from samples of mouse (Mus musculus) prefrontal cortices was obtained from GSE111708.

2 Pipeline combination details

2.1 Quality Control and Trimming

The bioinformatics pipeline begins by trimming raw reads from sequencing, which are stored in FASTQ files. Quality control is first conducted on the raw data using FastQC (Brown et al. 2017), which provides reports on base quality, contaminant presence, and sequence composition biases, helping to identify issues for correction before trimming. We tested three trimming tools to enhance read quality: BBduk, Trimmomatic, and Cutadapt. Each tool offers distinct features. BBduk (Whitham 2021) is optimized for high-speed performance, particularly on systems with large RAM, using k-mers for efficient contaminant and adapter removal and excelling at parallelized tasks. Trimmomatic (Bolger et al. 2014) is slightly slower but highly flexible, with sliding windows for adaptive trimming, though its sequential processing can extend execution times. Cutadapt (Martin 2011) balances speed and precision, is great for specific adapter removal but requires adequate memory for best performance. Post-trimming, each tool produces higher-quality data, yielding shorter, more accurate reads ready for alignment.

2.2 Alignment

Cleaned reads obtained for each sample were aligned (or "mapped") to the rat reference genome. In this study, we used STAR (Dobin et al. 2013), HiSat2 (Kim et al. 2015), and TopHat2 (Kim et al. 2013) to perform the alignment. STAR's compacted suffix graph algorithm enables rapid and precise splice junction detection, ideal for high-throughput projects. HiSat2 improves computational efficiency through hierarchical indexing and optimized memory usage, while TopHat2, historically significant for splice junction detection with Bowtie (Langmead et al. 2009), remains useful for certain transcriptome studies. The alignments generate SAM or BAM files, indicating each read's precise position on the reference.

2.3 Counting

After aligning reads to the reference, the next step is read counting, which quantifies reads mapped to each gene or transcript to assess gene abundance across samples. In this study, we tested HTSeq (Anders et al. 2015), StringTie (Pertea et al. 2015), and Cufflinks (Trapnell et al. 2010). HTSeq provides robust, simple read counting with 'Union' mode for broad coverage and 'Intersect' mode for high precision. StringTie excels in assembling and quantifying transcripts, especially in complex genomic regions, using a flow network algorithm for efficient transcript reconstruction. Cufflinks, an early tool for transcript quantification, remains useful for comparing new and historical datasets, though often replaced by StringTie in modern applications. This step produces a raw count expression matrix, which requires normalization to correct biases for accurate cross-sample comparisons.

2.4 Normalization

The normalization step is essential to adjust count data, eliminating biases from technical variations like sequencing depth. Without normalization, raw counts reflect only the number

of reads aligned to each gene, risking inaccurate sample comparisons. For this study, we tested the following normalization methods: TMM (Robinson and Oshlack 2010), FPKM (Trapnell et al. 2010), TPM (Wagner et al. 2012), and RLE (Anders and Huber 2010). Coverage adjusts counts based on sequencing coverage, while TMM corrects for library composition differences. FPKM and TPM normalize by transcript length and sequencing depth, with TPM preferred for inter-sample comparisons due to its direct comparability. RLE minimizes technical variation by adjusting expression levels relative to the sample median, ensuring that observed differences reflect true biological variations. These methods collectively support accurate, unbiased data interpretation across samples.

3 Corchete *et al*'s Pipeline precision and accuracy analysis

3.1 Precision Analysis

The first metric we used focuses on evaluating the variability of results within the control samples by analyzing a set of n subjects. For this analysis, we considered the genes expressed in these n control samples and calculated each gene's coefficient of variation (CoV). For each gene, the CoV, a measure of relative variability, is defined as follows:

$$\text{CoV} = \frac{\text{MAD}}{\text{Median}}$$

where the Median represents the median expression values of the gene for the n samples, and the MAD (Median Absolute Deviation) is calculated by:

$$\text{MAD} = \text{median}(|X_i - \text{Median}(X)|) \quad \text{for } i \in \{1, 2, \ldots, n\}$$

with $X = \{X_1, \ldots, X_n\}$ where X_i is the expression of the gene in the i-th sample. This non-parametric method was chosen because it allows for measuring data variability independently of measurement units. For more details on this approach, refer to Corchete *et al.* (Corchete et al. 2020).

For all genes, the CoV was calculated within each pipeline. The median CoV rank for each pipeline was determined, and this value was used as an indicator of pipeline variability (or dispersion). A lower rank index indicates better pipeline stability, meaning lower variability of obtained results. This procedure is graphically illustrated in Figure 3.

3.2 Accuracy Analysis

To assess the performance of the RNA-Seq pipelines, a detailed analysis was performed on p genes evaluated across n control samples using correlation. This critical metric determines how well sequencing data aligns with reference methods like qRT-PCR. We calculated the performance of each pipeline based on the expression values of genes obtained by qRT-PCR, a method known for its accuracy and sensitivity. Pearson's correlation coefficient (r) was used to measure the association between RNA-Seq and qRT-PCR data. The correlation coefficients r were calculated using the `corr.test` function from the R package `psych` (Revelle 2015). Once

these coefficients were calculated, a ranking (based on their decreasing order) of each gene according to each pipeline was established.

This ranking allows the prioritization of pipelines based on their accuracy for each specific gene. Then, a median ranking was determined, providing an overall measure of the pipeline's performance across all analyzed genes. The lower the median rank, the more accurate and reliable the pipeline is deemed. This procedure is graphically illustrated in Figure 3, where pipelines are compared based on their median ranking. Thus, the approach we adopted Thus compares the pipelines according to their consistency with the reference method qRT-PCR. Here again, a lower rank index indicates a better pipeline.

4 Proposed Pipeline analysis based on classifiers

In bioinformatics, the most reliable results are often obtained by applying machine learning techniques after feature selection or dimensionality reduction. Following this principle, we retained the 16,000 variables with the highest variance in each dataset. As the study was conducted in a weak-signal setting, a large number of variables were retained. This approach allows us to capture as much information as possible while ensuring that machine learning methods are applied accurately.

4.1 Classification Algorithms

4.1.1 Random Forest

Random forests (RF) are an ensemble of decision trees (Breiman 2001). The number of trees grown in each forest was fixed to 500, and the number of features randomly selected at each tree node had to be chosen. Several experiments were conducted to optimize this parameter. Optimization was achieved using Leave-One-Out Cross-Validation (LOOCV) (Kohavi 1995). For each pipeline, we compared these performances to identify the best-performing pipelines in terms of classification ability for samples across experimental conditions.

4.1.2 Support Vector Machines

We also evaluated the classification ability of bioinformatics pipelines using Support Vector Machines (SVM) with two types of kernels: a linear kernel and a Gaussian kernel (RBF, Radial Basis Function). SVMs are particularly well-suited for binary and multiclass classification problems, where the goal is to find the hyperplane that separates different classes with maximum margin. For each pipeline, SVMs with both linear and Gaussian kernels were evaluated using LOOCV, similar to the method used for random forests.

4.2 Classification Metric

Several measures are available to assess classification performance, including sensitivity, specificity, accuracy and Area Under the Curve (AUC). In this study, Normalized Mutual Information (NMI) was chosen as it is the primary metric to evaluate the performance of RNA-Seq pipelines due to its superiority in contexts where relationships between variables are complex (Kachouie

and Shutaywi 2020). Indeed, compared to accuracy, which can be inadequate for evaluating models in contexts where classes are unbalanced, or relationships between variables are nonlinear, NMI provides a more robust and informative measure (Kachouie and Shutaywi 2020). To obtain rankings that are more informed than those based on a single metric, we combined the unsupervised clustering metric NMI, and the F1-score—a supervised classification metric. NMI helps mitigate label-assignment errors that can occur under subtle signal conditions, and combining both metrics strengthens the evaluation by integrating two complementary perspectives.

4.3 Top Ranking Pipeline Selection

Initially, the ranking was established for each classifier by assigning rank 1 to the one with the highest NMI value. Thus, each classifier (Random Forest, SVM with linear and Gaussian kernel) was evaluated individually based on the NMI metric. The obtained ranks were then summed for each pipeline, allowing the calculation of an overall score. The pipeline with the lowest sum of ranks was designated as the best performing. The same applies to the score F1.

Based on the sum of both ranks metrics, this approach adheres to the principle of fair weighting of classifier performance. It thus makes for a rigorous and balanced evaluation, ensuring that the selected pipelines are distinguished by their stability across the different classifiers. Where the merging of rankings resulted in ties, an additional ranking step was added based on the effect size of differential gene expression (DGE) to break the ties. Although DGE is commonly used as a reference in RNA-Seq data analyses, we deliberately avoided relying on it as a primary ranking strategy. When viewed through the lens of differentially expressed genes, DGE inherently presents two extremes: very few DGEs, which increases the risk of false negatives, or an excessive number of DGEs, which increases the risk of false positives. For this reason, we used DGE only as a secondary criterion, ensuring a more balanced variable selection process.

5 Supplementary Figures

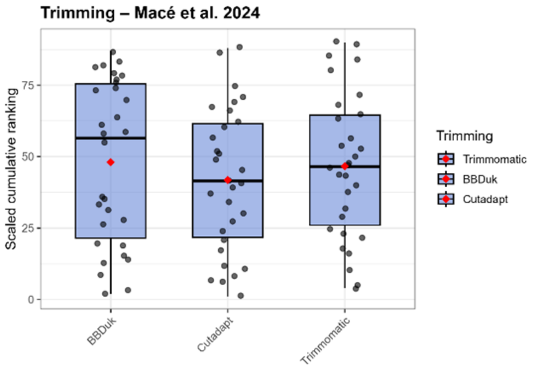

Trimming algorithms

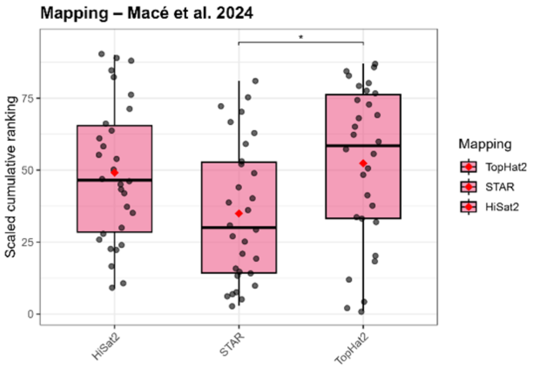

Alignment algorithms

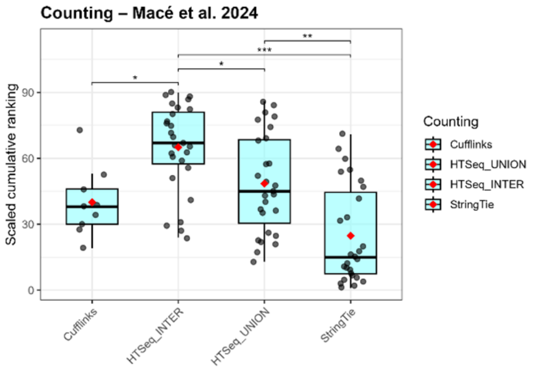

Counting methods

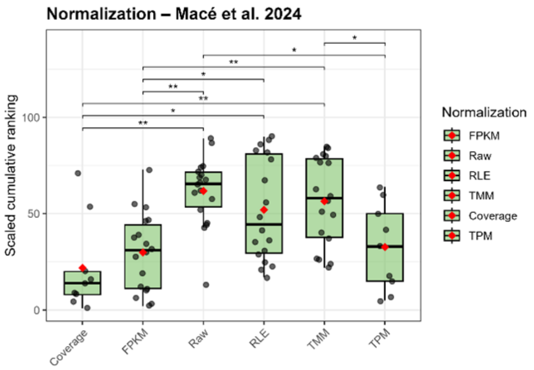

Normalization methods

Supp Fig. 1: Influence of the algorithms on the RNA-seq raw gene expression quantification. Box-plot analysis of the 90 pipelines grouped by the algorithms used at each step of the procedure: (a) trimming algorithms, (b) alignment algorithms, (c) counting methods, and (d) normalization methods. The red diamond represents the mean of the ranking reached by the pipelines that use the respective method or algorithm. Asterisks indicate the significance of the post-hoc Dunn's test: *p $<$ 0.05, **p $<$ 0.01, and ***p $<$ 0.001. Comparisons without an asterisk are statistically insignificant (p $>$ 0.05).

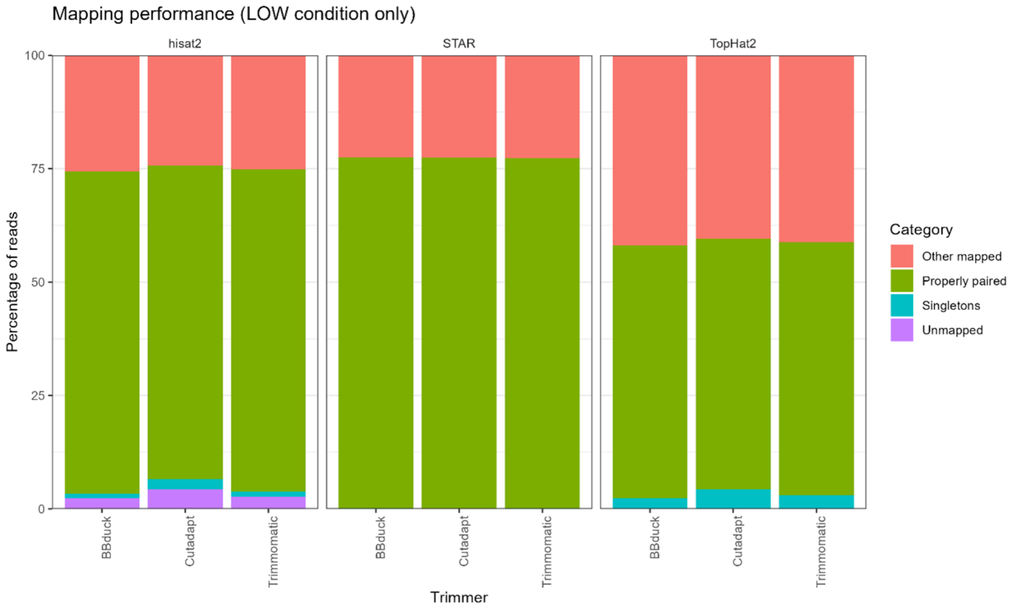

Supp Fig. 2: Mapping performance across trimmer–mapper combinations for the LOW-condition samples on the Macé *et al.* dataset. The stacked barplot summarizes the proportions of properly paired reads, singletons, other mapped reads, and unmapped reads obtained for each combination of trimming algorithm (BBDuk, Cutadapt, Trimmomatic) and alignment method (HiSat2, STAR, TopHat2). Percentages were computed from SAMtools `flagstat` outputs. This analysis provides a descriptive overview of alignment behaviour across preprocessing choices in the LOW-condition subset.

6 Supplementary Tables

Supp Table 1: NMI values for Random Forest (RF), SVM with Gaussian kernel (SVM.G), and SVM with linear kernel (SVM.L) for each pipeline on the Macé *et al.* dataset.

Pipeline	RF	SVM.G	SVM.L	Rank RF	Rank SVM.G	Rank SVM.L	Sum Rank	Rank
Trimmomatic_TopHat2 _Stringtie_Coverage	0,6588	0,883	0,8181	1	1	3	5	1
Cutadapt_STAR _Stringtie_FPKM	0,6254	0,883	0,883	8	1	1	10	2
Cutadapt_STAR _Stringtie_TPM	0,6254	0,883	0,883	8	1	1	10	3
BBduk_STAR _Stringtie_FPKM	0,6254	0,883	0,8181	8	1	3	12	4
Trimmomatic_STAR _Stringtie_Coverage	0,6254	0,883	0,8181	8	1	3	12	5
Cutadapt_STAR _Stringtie_Coverage	0,6254	0,8181	0,8181	8	8	3	19	6

Continued on next page

Pipeline	RF	SVM.G	SVM.L	Rank RF	Rank SVM.G	Rank SVM.L	Sum Rank	Rank
Trimmomatic_STAR _Stringtie_TPM	0,6254	0,8181	0,8181	8	8	3	19	7
BBduk_TopHat2 _Stringtie_FPKM	0,6254	0,883	0,7714	8	1	14	23	8
BBduk_STAR _Stringtie_Coverage	0,6254	0,7714	0,8181	8	13	3	24	9
BBduk_TopHat2 _Stringtie_TPM	0,6588	0,7052	0,8181	1	21	3	25	10
BBduk_TopHat2 _Stringtie_Coverage	0,6588	0,8181	0,7062	1	8	17	26	11
Cutadapt_TopHat2 _Stringtie_FPKM	0,6588	0,7714	0,7714	1	13	14	28	12
Trimmomatic_TopHat2 _Stringtie_FPKM	0,6254	0,712	0,8181	8	18	3	29	13
BBduk_STAR _Stringtie_TPM	0,6254	0,7714	0,7724	8	13	12	33	14
Cutadapt_TopHat2 _Stringtie_Coverage	0,6588	0,8181	0,5608	1	8	25	34	15
Trimmomatic_STAR _Stringtie_FPKM	0,6254	0,7714	0,7714	8	13	14	35	16
Cutadapt_TopHat2 _Stringtie_TPM	0,6588	0,4805	0,8181	1	49	3	53	17
Trimmomatic_TopHat2 _Stringtie_TPM	0,6588	0,4806	0,7724	1	48	12	61	18
Trimmomatic_hisat2 _Stringtie_FPKM	0,3855	0,8181	0,6588	37	8	19	64	19
Cutadapt_hisat2 _HTSeq_U_Raw	0,4883	0,7062	0,3855	19	19	42	80	20
Cutadapt_hisat2 _Stringtie_FPKM	0,3404	0,883	0,6254	65	1	20	86	21
BBduk_hisat2 _Stringtie_FPKM	0,3855	0,5405	0,6254	37	31	20	88	22
Trimmomatic_hisat2 _HTSeq_U_RLE	0,4382	0,4837	0,443	20	36	37	93	23
Cutadapt_STAR _HTSeq_IN_TMM	0,4382	0,4869	0,3843	20	35	46	101	24
Trimmomatic_STAR _HTSeq_U_RLE	0,4382	0,5447	0,3372	20	28	54	102	25

Continued on next page

Pipeline	RF	SVM.G	SVM.L	Rank RF	Rank SVM.G	Rank SVM.L	Sum Rank	Rank
BBduk_STAR _HTSeq_IN_RLE	0,4382	0,4773	0,4536	20	51	32	103	26
Cutadapt_hisat2 _HTSeq_IN_TMM	0,4382	0,4892	0,3546	20	33	50	103	27
Cutadapt_hisat2 _HTSeq_U_TMM	0,4085	0,4892	0,4161	33	33	39	105	28
BBduk_hisat2 _HTSeq_U_TMM	0,3818	0,5436	0,4161	39	29	39	107	29
Cutadapt_TopHat2 _HTSeq_IN_RLE	0,4382	0,4814	0,3546	20	38	50	108	30
Cutadapt_hisat2 _HTSeq_U_RLE	0,3818	0,4814	0,4536	39	38	32	109	31
Cutadapt_STAR _HTSeq_U_RLE	0,3818	0,4814	0,4536	39	38	32	109	32
BBduk_hisat2 _Stringtie_Coverage	0,3404	0,6593	0,5227	65	23	27	115	33
Trimmomatic_STAR _HTSeq_IN_RLE	0,3782	0,4814	0,4536	46	38	32	116	34
Trimmomatic_STAR _HTSeq_U_TMM	0,4382	0,4822	0,3354	20	37	63	120	35
Cutadapt_hisat2 _Stringtie_TPM	0,2989	0,7676	0,5644	83	17	23	123	36
Cutadapt_STAR _HTSeq_U_TMM	0,4382	0,4919	0,3042	20	32	71	123	37
BBduk_hisat2 _HTSeq_U_Raw	0,4085	0,5436	0,3354	33	29	63	125	38
Trimmomatic_TopHat2 _HTSeq_U_RLE	0,3782	0,4814	0,3855	46	38	42	126	39
BBduk_hisat2 _Stringtie_TPM	0,2989	0,7037	0,6029	83	22	22	127	40
Cutadapt_hisat2 _Stringtie_Coverage	0,2989	0,6593	0,5644	83	23	23	129	41
Cutadapt_STAR _HTSeq_U_Raw	0,3592	0,4814	0,4892	62	38	30	130	42
Cutadapt_TopHat2 _HTSeq_IN_TMM	0,3818	0,4814	0,3372	39	38	54	131	43
BBduk_STAR _HTSeq_U_Raw	0,3818	0,4356	0,4027	39	53	41	133	44

Continued on next page

Pipeline	RF	SVM.G	SVM.L	Rank RF	Rank SVM.G	Rank SVM.L	Sum Rank	Rank
Trimmomatic_hisat2 _Stringtie_TPM	0,2831	0,641	0,6611	89	26	18	133	45
Trimmomatic_STAR _HTSeq_U_Raw	0,4085	0,4814	0,3354	33	38	63	134	46
BBduk_hisat2 _HTSeq_IN_Raw	0,4356	0,4382	0,3372	30	52	54	136	47
Cutadapt_hisat2 _HTSeq_IN_Raw	0,3782	0,4278	0,5436	46	64	26	136	48
Trimmomatic_hisat2 _Stringtie_Coverage	0,2989	0,6458	0,5001	83	25	29	137	49
Trimmomatic_TopHat2 _Cufflinks_FPKM	0,39	0,4356	0,3818	36	53	48	137	50
Trimmomatic_TopHat2 _HTSeq_IN_RLE	0,3782	0,4814	0,3372	46	38	54	138	51
Cutadapt_STAR _HTSeq_IN_RLE	0,4382	0,4136	0,3372	20	69	54	143	52
Trimmomatic_hisat2 _HTSeq_U_TMM	0,3592	0,7062	0,3327	62	19	69	150	53
BBduk_TopHat2 _Cufflinks_FPKM	0,3738	0,4356	0,4368	61	53	38	152	54
BBduk_hisat2 _HTSeq_U_RLE	0,3818	0,4814	0,2928	39	38	80	157	55
Cutadapt_STAR _HTSeq_IN_Raw	0,3782	0,4268	0,3843	46	65	46	157	56
Trimmomatic_STAR _HTSeq_IN_Raw	0,3782	0,3738	0,4892	46	82	30	158	57
BBduk_hisat2 _HTSeq_IN_TMM	0,3782	0,3738	0,4536	46	82	32	160	58
Cutadapt_hisat2 _Cufflinks_FPKM	0,4382	0,4356	0,2193	20	53	87	160	59
BBduk_STAR _HTSeq_IN_Raw	0,3404	0,5528	0,3042	65	27	71	163	60
Cutadapt_TopHat2 _Cufflinks_FPKM	0,4238	0,4356	0,2794	32	53	83	168	61
BBduk_TopHat2 _HTSeq_IN_TMM	0,3782	0,3855	0,3546	46	73	50	169	62
Trimmomatic_STAR _Cufflinks_FPKM	0,4356	0,4356	0,219	30	53	88	171	63

Continued on next page

Pipeline	RF	SVM.G	SVM.L	Rank RF	Rank SVM.G	Rank SVM.L	Sum Rank	Rank
Cutadapt_TopHat2 _HTSeq_U_RLE	0,3404	0,4268	0,3855	65	65	42	172	64
BBduk_STAR _HTSeq_IN_TMM	0,3291	0,4136	0,5112	78	69	28	175	65
Trimmomatic_hisat2 _Cufflinks_FPKM	0,3782	0,4356	0,281	46	53	82	181	66
Cutadapt_STAR _Cufflinks_FPKM	0,3782	0,4356	0,2339	46	53	85	184	67
Trimmomatic_hisat2 _HTSeq_U_Raw	0,3404	0,4794	0,3042	65	50	71	186	68
BBduk_hisat2 _Cufflinks_FPKM	0,3782	0,4356	0,191	46	53	89	188	69
BBduk_STAR _Cufflinks_FPKM	0,3791	0,4356	0,1812	45	53	90	188	70
BBduk_TopHat2 _HTSeq_IN_RLE	0,3782	0,3354	0,3372	46	88	54	188	71
BBduk_hisat2 _HTSeq_IN_RLE	0,3782	0,3006	0,3372	46	90	54	190	72
BBduk_TopHat2 _HTSeq_U_RLE	0,3404	0,3855	0,3372	65	73	54	192	73
Cutadapt_TopHat2 _HTSeq_U_TMM	0,3592	0,3738	0,3546	62	82	50	194	74
Trimmomatic_hisat2 _HTSeq_IN_TMM	0,3291	0,4136	0,3772	78	69	49	196	75
Trimmomatic_TopHat2 _HTSeq_IN_TMM	0,3782	0,3354	0,3354	46	88	63	197	76
BBduk_STAR _HTSeq_U_RLE	0,3782	0,3738	0,3042	46	82	71	199	77
Cutadapt_TopHat2 _HTSeq_IN_Raw	0,3404	0,4268	0,3042	65	65	71	201	78
Trimmomatic_STAR _HTSeq_IN_TMM	0,3404	0,4268	0,3042	65	65	71	201	79
Cutadapt_hisat2 _HTSeq_IN_RLE	0,3291	0,3738	0,3855	78	82	42	202	80
BBduk_STAR _HTSeq_U_TMM	0,3291	0,3855	0,3372	78	73	54	205	81
Trimmomatic_Tophat2 _HTSeq_U_Raw	0,3404	0,3791	0,3129	65	78	70	213	82

Continued on next page

Pipeline	RF	SVM.G	SVM.L	Rank RF	Rank SVM.G	Rank SVM.L	Sum Rank	Rank
BBduk_TopHat2 _HTSeq_U_TMM	0,3291	0,3855	0,3354	78	73	63	214	83
Trimmomatic_hisat2 _HTSeq_IN_RLE	0,3404	0,3907	0,2994	65	72	79	216	84
Trimmomatic_TopHat2 _HTSeq_U_TMM	0,3404	0,3772	0,3042	65	80	71	216	85
BBduk_TopHat2 _HTSeq_IN_Raw	0,3404	0,3738	0,3042	65	82	71	218	86
Trimmomatic_hisat2 _HTSeq_IN_Raw	0,2989	0,3855	0,3354	83	73	63	219	87
Trimmomatic_Tophat2 _HTSeq_IN_Raw	0,3404	0,3772	0,2863	65	80	81	226	88
Cutadapt_TopHat2 _HTSeq_U_Raw	0,2764	0,4356	0,2305	90	53	86	229	89
BBduk_TopHat2 _HTSeq_U_Raw	0,2989	0,3791	0,2411	83	78	84	245	90

Supp Table 2: F1-score values for Random Forest (RF), SVM with Gaussian kernel (SVM.G), and SVM with linear kernel (SVM.L) for each pipeline on the Macé *et al.* dataset.

Pipeline	RF	SVM.G	SVM.L	Rank RF	Rank SVM.G	Rank SVM.L	Sum Rank	Rank
Cutadapt_TopHat2 _Stringtie_Coverage	0,8333	0,9153	0,4667	1	12	34	47	1
Trimmomatic_STAR _Cufflinks_FPKM	0,7912	0,7912	0,6144	9	41	1	51	2
BBduk_STAR _Cufflinks_FPKM	0,752	0,7912	0,5402	23	41	6	70	3
BBduk_STAR _Stringtie_TPM	0,7908	0,9167	0,4386	11	8	51	70	4
Cutadapt_TopHat2 _Stringtie_FPKM	0,8333	0,9167	0,4167	1	8	63	72	5
Cutadapt_hisat2 _Stringtie_FPKM	0,7028	0,9582	0,5399	65	1	7	73	6
Trimmomatic_TopHat2 _Stringtie_Coverage	0,8333	0,9582	0,4074	1	1	71	73	7
BBduk_TopHat2 _Stringtie_FPKM	0,7908	0,9582	0,4167	11	1	63	75	8

Continued on next page

Pipeline	RF	SVM.G	SVM.L	Rank RF	Rank SVM.G	Rank SVM.L	Sum Rank	Rank
Cutadapt_STAR _Cufflinks_FPKM	0,7481	0,7912	0,5846	34	41	2	77	9
Trimmomatic_hisat2 _Cufflinks_FPKM	0,7481	0,7912	0,5611	34	41	4	79	10
Trimmomatic_STAR _Stringtie_FPKM	0,7908	0,9167	0,4167	11	8	63	82	11
BBduk_STAR _Stringtie_FPKM	0,7908	0,9582	0,4074	11	1	71	83	12
Trimmomatic_STAR _Stringtie_Coverage	0,7908	0,9582	0,4074	11	1	71	83	13
BBduk_hisat2 _Cufflinks_FPKM	0,7481	0,7912	0,528	34	41	9	84	14
Cutadapt_STAR _HTSeq_U_TMM	0,7495	0,7917	0,4874	24	40	22	86	15
BBduk_STAR _HTSeq_IN_RLE	0,7495	0,795	0,4652	24	29	35	88	16
BBduk_STAR _Stringtie_Coverage	0,7908	0,9167	0,4074	11	8	71	90	17
BBduk_hisat2 _HTSeq_U_TMM	0,7127	0,8296	0,5165	51	26	16	93	18
Trimmomatic_TopHat2 _HTSeq_IN_RLE	0,7481	0,7926	0,4739	34	30	29	93	19
Cutadapt_STAR _Stringtie_Coverage	0,7908	0,9153	0,4074	11	12	71	94	20
Trimmomatic_STAR _Stringtie_TPM	0,7908	0,9153	0,4074	11	12	71	94	21
BBduk_hisat2 _Stringtie_Coverage	0,7028	0,8721	0,5266	65	21	10	96	22
BBduk_STAR _HTSeq_U_Raw	0,7127	0,7912	0,5557	51	41	5	97	23
BBduk_TopHat2 _Stringtie_Coverage	0,8333	0,9153	0,3852	1	12	85	98	24
Cutadapt_hisat2 _Cufflinks_FPKM	0,7495	0,7912	0,4693	24	41	33	98	25
Trimmomatic_STAR _HTSeq_IN_RLE	0,7481	0,7926	0,4652	34	30	35	99	26
BBduk_hisat2 _HTSeq_U_RLE	0,7127	0,7926	0,5018	51	30	19	100	27

Continued on next page

Pipeline	RF	SVM.G	SVM.L	Rank RF	Rank SVM.G	Rank SVM.L	Sum Rank	Rank
Cutadapt_STAR _Stringtie_FPKM	0,7908	0,9582	0,3725	11	1	88	100	28
Cutadapt_STAR _Stringtie_TPM	0,7908	0,9582	0,3725	11	1	88	100	29
Trimmomatic_TopHat2 _Stringtie_FPKM	0,7908	0,8745	0,4074	11	18	71	100	30
Cutadapt_TopHat2 _HTSeq_IN_RLE	0,7495	0,7926	0,4398	24	30	47	101	31
Trimmomatic_STAR _HTSeq_U_RLE	0,7495	0,8328	0,4369	24	24	53	101	32
Trimmomatic_hisat2 _HTSeq_U_RLE	0,7495	0,789	0,4741	24	52	28	104	33
Cutadapt_hisat2 _HTSeq_U_Raw	0,7936	0,8741	0,4023	8	19	80	107	34
BBduk_STAR _HTSeq_U_RLE	0,7481	0,7474	0,5266	34	65	10	109	35
Cutadapt_TopHat2 _HTSeq_IN_TMM	0,7127	0,7926	0,4739	51	30	29	110	36
Trimmomatic_hisat2 _Stringtie_Coverage	0,6667	0,832	0,5721	83	25	3	111	37
BBduk_hisat2 _HTSeq_IN_Raw	0,7912	0,7467	0,4739	9	75	29	113	38
Cutadapt_STAR _HTSeq_IN_RLE	0,7495	0,751	0,4739	24	60	29	113	39
Cutadapt_hisat2 _HTSeq_U_RLE	0,7127	0,7926	0,4652	51	30	35	116	40
Cutadapt_STAR _HTSeq_U_RLE	0,7127	0,7926	0,4652	51	30	35	116	41
Trimmomatic_hisat2 _HTSeq_U_TMM	0,6686	0,8741	0,5055	80	19	18	117	42
Trimmomatic_hisat2 _Stringtie_FPKM	0,6655	0,9153	0,5	87	12	20	119	43
Cutadapt_hisat2 _Stringtie_TPM	0,6667	0,9132	0,4761	83	17	26	126	44
Cutadapt_hisat2 _HTSeq_IN_TMM	0,7495	0,7844	0,4398	24	56	47	127	45
BBduk_hisat2 _HTSeq_U_Raw	0,7037	0,8296	0,4467	62	26	40	128	46

Continued on next page

Pipeline	RF	SVM.G	SVM.L	Rank RF	Rank SVM.G	Rank SVM.L	Sum Rank	Rank
BBduk_STAR _HTSeq_IN_Raw	0,7028	0,7859	0,5266	65	54	10	129	47
Cutadapt_hisat2 _Stringtie_Coverage	0,6667	0,8721	0,4761	83	21	26	130	48
Cutadapt_TopHat2 _Cufflinks_FPKM	0,7563	0,7912	0,4087	22	41	67	130	49
Trimmomatic_hisat2 _HTSeq_U_Raw	0,7028	0,7855	0,5266	65	55	10	130	50
Trimmomatic_STAR _HTSeq_U_Raw	0,7037	0,7926	0,4467	62	30	40	132	51
BBduk_hisat2 _HTSeq_IN_TMM	0,7481	0,7474	0,4652	34	65	35	134	52
Cutadapt_hisat2 _HTSeq_U_TMM	0,7037	0,7844	0,5165	62	56	16	134	53
Trimmomatic_TopHat2 _Stringtie_TPM	0,8333	0,7155	0,4386	1	84	51	136	54
Cutadapt_TopHat2 _HTSeq_IN_Raw	0,7028	0,7474	0,5266	65	65	10	140	55
Trimmomatic_Tophat2 _HTSeq_IN_Raw	0,7028	0,7479	0,5252	65	63	15	143	56
Trimmomatic_Tophat2 _HTSeq_U_Raw	0,7028	0,752	0,4884	65	58	21	144	57
Trimmomatic_TopHat2 _HTSeq_U_RLE	0,7481	0,7926	0,4023	34	30	80	144	58
Cutadapt_STAR _HTSeq_IN_TMM	0,7495	0,7871	0,4078	24	53	69	146	59
Trimmomatic_STAR _HTSeq_U_TMM	0,7495	0,7382	0,4467	24	82	40	146	60
BBduk_hisat2 _Stringtie_TPM	0,6711	0,8664	0,4415	78	23	46	147	61
Trimmomatic_TopHat2 _HTSeq_U_TMM	0,7028	0,7479	0,4874	65	63	22	150	62
BBduk_TopHat2 _HTSeq_IN_Raw	0,7028	0,7474	0,4874	65	65	22	152	63
Trimmomatic_STAR _HTSeq_IN_TMM	0,7028	0,7474	0,4874	65	65	22	152	64
Trimmomatic_TopHat2 _Cufflinks_FPKM	0,7163	0,7912	0,4254	50	41	61	152	65

Continued on next page

Pipeline	RF	SVM.G	SVM.L	Rank RF	Rank SVM.G	Rank SVM.L	Sum Rank	Rank
BBduk_TopHat2 _HTSeq_IN_TMM	0,7481	0,7467	0,4398	34	75	47	156	66
Cutadapt_TopHat2 _Stringtie_TPM	0,8333	0,7121	0,4074	1	85	71	157	67
Trimmomatic_STAR _HTSeq_IN_Raw	0,7481	0,7474	0,4272	34	65	59	158	68
Trimmomatic_TopHat2 _HTSeq_IN_TMM	0,7481	0,7051	0,4467	34	86	40	160	69
BBduk_TopHat2 _Stringtie_TPM	0,8333	0,6393	0,4074	1	90	71	162	70
Cutadapt_STAR _HTSeq_IN_Raw	0,7481	0,7474	0,4078	34	65	69	168	71
Cutadapt_STAR _HTSeq_U_Raw	0,6686	0,7926	0,4272	80	30	59	169	72
BBduk_TopHat2 _HTSeq_U_TMM	0,7103	0,7467	0,4467	57	75	40	172	73
BBduk_TopHat2 _HTSeq_IN_RLE	0,7481	0,7051	0,4369	34	86	53	173	74
BBduk_STAR _HTSeq_IN_TMM	0,7103	0,751	0,4366	57	60	58	175	75
BBduk_hisat2 _HTSeq_IN_RLE	0,7481	0,6581	0,4369	34	89	53	176	76
BBduk_hisat2 _Stringtie_FPKM	0,6655	0,726	0,5399	87	83	7	177	77
BBduk_TopHat2 _Cufflinks_FPKM	0,7474	0,7912	0,3719	49	41	90	180	78
Trimmomatic_hisat2 _Stringtie_TPM	0,6201	0,8148	0,4248	90	28	62	180	79
BBduk_STAR _HTSeq_U_TMM	0,7103	0,7467	0,4369	57	75	53	185	80
Cutadapt_TopHat2 _HTSeq_U_TMM	0,6686	0,7474	0,4398	80	65	47	192	81
BBduk_TopHat2 _HTSeq_U_RLE	0,7028	0,7467	0,4369	65	75	53	193	82
Cutadapt_TopHat2 _HTSeq_U_Raw	0,6294	0,7912	0,4167	89	41	63	193	83
Trimmomatic_hisat2 _HTSeq_IN_Raw	0,6667	0,7467	0,4467	83	75	40	198	84

Continued on next page

Pipeline	RF	SVM.G	SVM.L	Rank RF	Rank SVM.G	Rank SVM.L	Sum Rank	Rank
Cutadapt_hisat2 _HTSeq_IN_Raw	0,7481	0,7389	0,3852	34	81	85	200	85
Trimmomatic_hisat2 _HTSeq_IN_TMM	0,7103	0,751	0,4006	57	60	84	201	86
Cutadapt_hisat2 _HTSeq_IN_RLE	0,7103	0,7474	0,4023	57	65	80	202	87
BBduk_TopHat2 _HTSeq_U_Raw	0,6711	0,752	0,4087	78	58	67	203	88
Cutadapt_TopHat2 _HTSeq_U_RLE	0,7028	0,7474	0,4023	65	65	80	210	89
Trimmomatic_hisat2 _HTSeq_IN_RLE	0,7028	0,6997	0,3833	65	88	87	240	90

Supp Table 3: NMI values for Random Forest (RF), SVM with Gaussian kernel (SVM.G), and SVM with linear kernel (SVM.L) for each pipeline on the Murat *et al.* dataset.

Pipeline	RF	SVM.G	SVM.L	Rank RF	Rank SVM.G	Rank SVM.L	Sum Rank	Rank
BBduk_hisat2 _Stringtie_FPKM	0,6423	0,612584	0,553136	3	1	2	6	1
Trimmomatic_hisat2 _Stringtie_TPM	0,6423	0,612584	0,553136	3	1	2	6	2
Trimmomatic_STAR _Stringtie_FPKM	0,6423	0,612584	0,553136	3	1	2	6	3
Trimmomatic_STAR _Stringtie_TPM	0,6423	0,612584	0,553136	3	1	2	6	4
Trimmomatic_TopHat2 _HTSeq_U_TMM	0,6423	0,612584	0,553136	3	1	2	6	5
Trimmomatic_TopHat2 _Stringtie_TPM	0,6423	0,612584	0,553136	3	1	2	6	6
Trimmomatic_hisat2 _Stringtie_FPKM	0,6423	0,553136	0,553136	3	18	2	23	7
Trimmomatic_TopHat2 _HTSeq_IN_RLE	0,6423	0,553136	0,553136	3	18	2	23	8
Trimmomatic_TopHat2 _Stringtie_FPKM	0,5843	0,612584	0,553136	34	1	2	37	9
Trimmomatic_hisat2 _HTSeq_U_TMM	0,5996	0,553136	0,553136	29	18	2	49	10

Continued on next page

Pipeline	RF	SVM.G	SVM.L	Rank RF	Rank SVM.G	Rank SVM.L	Sum Rank	Rank
BBduk_TopHat2 _Stringtie_Coverage	0,6678	0,612584	0,529559	1	1	52	54	11
Trimmomatic_hisat2 _HTSeq_IN_TMM	0,5843	0,553136	0,553136	34	18	2	54	12
Trimmomatic_hisat2 _HTSeq_U_RLE	0,5843	0,553136	0,553136	34	18	2	54	13
Trimmomatic_STAR _HTSeq_U_TMM	0,5843	0,553136	0,553136	34	18	2	54	14
Trimmomatic_TopHat2 _HTSeq_IN_TMM	0,5843	0,553136	0,553136	34	18	2	54	15
Trimmomatic_TopHat2 _HTSeq_U_RLE	0,5843	0,553136	0,553136	34	18	2	54	16
Trimmomatic_TopHat2 _Stringtie_Coverage	0,6678	0,612584	0,529559	1	1	52	54	17
Cutadapt_TopHat2 _Stringtie_Coverage	0,6423	0,612584	0,529559	3	1	52	56	18
BBduk_hisat2 _Stringtie_TPM	0,6423	0,499511	0,553136	3	66	2	71	19
BBduk_STAR _Stringtie_TPM	0,6423	0,499511	0,553136	3	66	2	71	20
BBduk_TopHat2 _Stringtie_TPM	0,6423	0,499511	0,553136	3	66	2	71	21
Cutadapt_hisat2 _Stringtie_FPKM	0,6423	0,499511	0,553136	3	66	2	71	22
Cutadapt_hisat2 _Stringtie_TPM	0,6423	0,499511	0,553136	3	66	2	71	23
Trimmomatic_hisat2 _HTSeq_IN_RLE	0,5798	0,612584	0,553136	69	1	2	72	24
Trimmomatic_hisat2 _HTSeq_IN_Raw	0,6423	0,553136	0,529559	3	18	52	73	25
BBduk_STAR _HTSeq_U_TMM	0,5996	0,528403	0,553136	29	46	2	77	26
BBduk_TopHat2 _HTSeq_IN_TMM	0,5996	0,528403	0,553136	29	46	2	77	27
Cutadapt_STAR _HTSeq_IN_TMM	0,5996	0,528403	0,553136	29	46	2	77	28
BBduk_TopHat2 _Stringtie_FPKM	0,6423	0,40497	0,553136	3	75	2	80	29

Continued on next page

Pipeline	RF	SVM.G	SVM.L	Rank RF	Rank SVM.G	Rank SVM.L	Sum Rank	Rank
Cutadapt_STAR _Stringtie_FPKM	0,6423	0,40497	0,553136	3	75	2	80	30
Cutadapt_STAR _Stringtie_TPM	0,6423	0,40497	0,553136	3	75	2	80	31
Cutadapt_TopHat2 _Stringtie_TPM	0,6423	0,40497	0,553136	3	75	2	80	32
BBduk_hisat2 _HTSeq_U_TMM	0,5843	0,528403	0,553136	34	46	2	82	33
BBduk_STAR _HTSeq_IN_RLE	0,5843	0,528403	0,553136	34	46	2	82	34
BBduk_TopHat2 _HTSeq_IN_RLE	0,5843	0,528403	0,553136	34	46	2	82	35
Cutadapt_TopHat2 _HTSeq_IN_TMM	0,5843	0,528403	0,553136	34	46	2	82	36
Cutadapt_hisat2 _HTSeq_IN_TMM	0,6423	0,398753	0,553136	3	80	2	85	37
BBduk_hisat2 _Stringtie_Coverage	0,5843	0,612584	0,529559	34	1	52	87	38
BBduk_STAR _Stringtie_Coverage	0,5843	0,612584	0,529559	34	1	52	87	39
Cutadapt_STAR _Stringtie_Coverage	0,5843	0,612584	0,529559	34	1	52	87	40
Trimmomatic_hisat2 _Stringtie_Coverage	0,5843	0,612584	0,529559	34	1	52	87	41
Trimmomatic_STAR _Stringtie_Coverage	0,5843	0,612584	0,529559	34	1	52	87	42
Trimmomatic_STAR _HTSeq_U_RLE	0,5798	0,553136	0,553136	69	18	2	89	43
Cutadapt_hisat2 _Stringtie_Coverage	0,4801	0,612584	0,612584	88	1	1	90	44
Trimmomatic_STAR _HTSeq_IN_RLE	0,5659	0,553136	0,553136	79	18	2	99	45
Trimmomatic_STAR _HTSeq_IN_TMM	0,5659	0,553136	0,553136	79	18	2	99	46
Trimmomatic_STAR _Cufflinks_FPKM	0,6423	0,553136	0,452433	3	18	80	101	47
BBduk_STAR _Stringtie_FPKM	0,5843	0,499511	0,553136	34	66	2	102	48

Continued on next page

**Pipeline**	**RF**	**SVM.G**	**SVM.L**	**Rank RF**	**Rank SVM.G**	**Rank SVM.L**	**Sum Rank**	**Rank**
BBduk_hisat2 _HTSeq_IN_Raw	0,5843	0,553136	0,529559	34	18	52	104	49
BBduk_hisat2 _HTSeq_U_Raw	0,5843	0,553136	0,529559	34	18	52	104	50
BBduk_STAR _HTSeq_IN_Raw	0,5843	0,553136	0,529559	34	18	52	104	51
BBduk_STAR _HTSeq_U_Raw	0,5843	0,553136	0,529559	34	18	52	104	52
BBduk_TopHat2 _HTSeq_IN_Raw	0,5843	0,553136	0,529559	34	18	52	104	53
BBduk_TopHat2 _HTSeq_U_Raw	0,5843	0,553136	0,529559	34	18	52	104	54
Cutadapt_STAR _HTSeq_U_Raw	0,5843	0,553136	0,529559	34	18	52	104	55
Cutadapt_STAR _HTSeq_IN_Raw	0,5843	0,553136	0,529559	34	18	52	104	56
Cutadapt_TopHat2 _HTSeq_IN_Raw	0,5843	0,553136	0,529559	34	18	52	104	57
Cutadapt_TopHat2 _HTSeq_U_Raw	0,5843	0,553136	0,529559	34	18	52	104	58
Trimmomatic_hisat2 _HTSeq_U_Raw	0,5843	0,553136	0,529559	34	18	52	104	59
Trimmomatic_STAR _HTSeq_IN_Raw	0,5843	0,553136	0,529559	34	18	52	104	60
Trimmomatic_STAR _HTSeq_U_Raw	0,5843	0,553136	0,529559	34	18	52	104	61
Trimmomatic_TopHat2 _HTSeq_IN_Raw	0,5843	0,553136	0,529559	34	18	52	104	62
Trimmomatic_TopHat2 _HTSeq_U_Raw	0,5843	0,553136	0,529559	34	18	52	104	63
Cutadapt_hisat2 _HTSeq_U_RLE	0,5843	0,398753	0,553136	34	80	2	116	64
BBduk_TopHat2 _HTSeq_U_RLE	0,5798	0,528403	0,553136	69	46	2	117	65
Cutadapt_STAR _HTSeq_U_RLE	0,5798	0,528403	0,553136	69	46	2	117	66
Cutadapt_STAR _HTSeq_U_TMM	0,5798	0,528403	0,553136	69	46	2	117	67

Continued on next page

Pipeline	RF	SVM.G	SVM.L	Rank RF	Rank SVM.G	Rank SVM.L	Sum Rank	Rank
Cutadapt_TopHat2 _HTSeq_U_RLE	0,5798	0,528403	0,553136	69	46	2	117	68
Cutadapt_TopHat2 _HTSeq_U_TMM	0,5798	0,528403	0,553136	69	46	2	117	69
Cutadapt_TopHat2 _HTSeq_IN_RLE	0,5798	0,528403	0,553136	69	46	2	117	70
BBduk_hisat2 _HTSeq_IN_RLE	0,5659	0,528403	0,553136	79	46	2	127	71
BBduk_hisat2 _HTSeq_IN_TMM	0,5659	0,528403	0,553136	79	46	2	127	72
BBduk_hisat2 _HTSeq_U_RLE	0,5659	0,528403	0,553136	79	46	2	127	73
BBduk_TopHat2 _HTSeq_U_TMM	0,5659	0,528403	0,553136	79	46	2	127	74
Cutadapt_STAR _HTSeq_IN_RLE	0,5659	0,528403	0,553136	79	46	2	127	75
Cutadapt_TopHat2 _Stringtie_FPKM	0,5996	0,499511	0,529559	29	66	52	147	76
Bbduk_hisat2 _Cufflinks_FPKM	0,6423	0,499511	0,452433	3	66	80	149	77
Cutadapt_hisat2 _HTSeq_U_TMM	0,5798	0,398753	0,553136	69	80	2	151	78
Cutadapt_STAR _Cufflinks_FPKM	0,6423	0,499511	0,435493	3	66	84	153	79
BBduk_STAR _Cufflinks_FPKM	0,6423	0,40497	0,452433	3	75	80	158	80
Trimmomatic_TopHat2 _Cufflinks_FPKM	0,6423	0,392453	0,362814	3	83	88	174	81
Cutadapt_TopHat2 _Cufflinks_FPKM	0,6423	0,362814	0,346531	3	85	89	177	82
BBduk_STAR _HTSeq_IN_TMM	0,5305	0,528403	0,529559	86	46	52	184	83
BBduk_STAR _HTSeq_U_RLE	0,5305	0,528403	0,529559	86	46	52	184	84
Cutadapt_hisat2 _Cufflinks_FPKM	0,5843	0,289709	0,452433	34	90	80	204	85
Cutadapt_hisat2 _HTSeq_IN_RLE	0,5798	0,392453	0,529559	69	83	52	204	86

Continued on next page

Pipeline	RF	SVM.G	SVM.L	Rank RF	Rank SVM.G	Rank SVM.L	Sum Rank	Rank
Trimmomatic_hisat2 _Cufflinks_FPKM	0,5843	0,362814	0,417527	34	85	85	204	87
BBduk_TopHat2 _Cufflinks_FPKM	0,5843	0,362814	0,201161	34	85	90	209	88
Cutadapt_hisat2 _HTSeq_IN_Raw	0,4801	0,362814	0,392453	88	85	86	259	89
Cutadapt_hisat2 _HTSeq_U_Raw	0,4801	0,362814	0,392453	88	85	86	259	90

Supp Table 4: F1-score values for Random Forest (RF), SVM with Gaussian kernel (SVM.G), and SVM with linear kernel (SVM.L) for each pipeline on the Murat *et al.* dataset.

Pipeline	RF	SVM.G	SVM.L	Rank RF	Rank SVM.G	Rank SVM.L	Sum Rank	Rank
BBduk_hisat2 _Stringtie_FPKM	0,6199	0,4479	0,4292	3	21	2	26	1
Trimmomatic_hisat2 _Stringtie_TPM	0,6199	0,4479	0,4292	3	21	2	26	2
Trimmomatic_STAR _Stringtie_FPKM	0,6199	0,4479	0,4292	3	21	2	26	3
Trimmomatic_STAR _Stringtie_TPM	0,6199	0,4479	0,4292	3	21	2	26	4
Trimmomatic_TopHat2 _HTSeq_U_TMM	0,6199	0,4479	0,4292	3	21	2	26	5
Trimmomatic_TopHat2 _Stringtie_TPM	0,6199	0,4479	0,4292	3	21	2	26	6
BBduk_hisat2 _HTSeq_U_TMM	0,5931	0,4495	0,4292	29	1	2	32	7
BBduk_STAR _HTSeq_IN_RLE	0,5931	0,4495	0,4292	29	1	2	32	8
BBduk_TopHat2 _HTSeq_IN_RLE	0,5931	0,4495	0,4292	29	1	2	32	9
Cutadapt_TopHat2 _HTSeq_IN_TMM	0,5931	0,4495	0,4292	29	1	2	32	10
Trimmomatic_hisat2 _Stringtie_FPKM	0,6199	0,4292	0,4292	3	38	2	43	11
Trimmomatic_TopHat2 _HTSeq_IN_RLE	0,6199	0,4292	0,4292	3	38	2	43	12

Continued on next page

Pipeline	RF	SVM.G	SVM.L	Rank RF	Rank SVM.G	Rank SVM.L	Sum Rank	Rank
Trimmomatic_TopHat2 _Stringtie_FPKM	0,5931	0,4479	0,4292	29	21	2	52	13
BBduk_hisat2 _HTSeq_IN_RLE	0,5729	0,4495	0,4292	64	1	2	67	14
BBduk_hisat2 _HTSeq_IN_TMM	0,5729	0,4495	0,4292	64	1	2	67	15
BBduk_hisat2 _HTSeq_U_RLE	0,5729	0,4495	0,4292	64	1	2	67	16
BBduk_TopHat2 _HTSeq_U_TMM	0,5729	0,4495	0,4292	64	1	2	67	17
Cutadapt_STAR _HTSeq_IN_RLE	0,5729	0,4495	0,4292	64	1	2	67	18
Trimmomatic_hisat2 _HTSeq_IN_TMM	0,5931	0,4292	0,4292	29	38	2	69	19
Trimmomatic_hisat2 _HTSeq_U_RLE	0,5931	0,4292	0,4292	29	38	2	69	20
Trimmomatic_STAR _HTSeq_U_TMM	0,5931	0,4292	0,4292	29	38	2	69	21
Trimmomatic_TopHat2 _HTSeq_IN_TMM	0,5931	0,4292	0,4292	29	38	2	69	22
Trimmomatic_TopHat2 _HTSeq_U_RLE	0,5931	0,4292	0,4292	29	38	2	69	23
Cutadapt_hisat2 _HTSeq_IN_TMM	0,6199	0,4248	0,4292	3	66	2	71	24
BBduk_STAR _HTSeq_U_TMM	0,5723	0,4495	0,4292	71	1	2	74	25
BBduk_TopHat2 _HTSeq_IN_TMM	0,5723	0,4495	0,4292	71	1	2	74	26
Cutadapt_STAR _HTSeq_IN_TMM	0,5723	0,4495	0,4292	71	1	2	74	27
BBduk_hisat2 _Stringtie_TPM	0,6199	0,42	0,4292	3	71	2	76	28
BBduk_STAR _Stringtie_TPM	0,6199	0,42	0,4292	3	71	2	76	29
BBduk_TopHat2 _Stringtie_Coverage	0,6379	0,4479	0,4077	1	21	54	76	30
BBduk_TopHat2 _Stringtie_TPM	0,6199	0,42	0,4292	3	71	2	76	31

Continued on next page

Pipeline	RF	SVM.G	SVM.L	Rank RF	Rank SVM.G	Rank SVM.L	Sum Rank	Rank
Cutadapt_hisat2 _Stringtie_FPKM	0,6199	0,42	0,4292	3	71	2	76	32
Cutadapt_hisat2 _Stringtie_TPM	0,6199	0,42	0,4292	3	71	2	76	33
Trimmomatic_TopHat2 _Stringtie_Coverage	0,6379	0,4479	0,4077	1	21	54	76	34
Cutadapt_TopHat2 _Stringtie_Coverage	0,6199	0,4479	0,4077	3	21	54	78	35
BBduk_TopHat2 _HTSeq_U_RLE	0,5485	0,4495	0,4292	79	1	2	82	36
Cutadapt_STAR _HTSeq_U_RLE	0,5485	0,4495	0,4292	79	1	2	82	37
Cutadapt_STAR _HTSeq_U_TMM	0,5485	0,4495	0,4292	79	1	2	82	38
Cutadapt_TopHat2 _HTSeq_U_RLE	0,5485	0,4495	0,4292	79	1	2	82	39
Cutadapt_TopHat2 _HTSeq_U_TMM	0,5485	0,4495	0,4292	79	1	2	82	40
Cutadapt_TopHat2 _HTSeq_IN_RLE	0,5485	0,4495	0,4292	79	1	2	82	41
BBduk_TopHat2 _Stringtie_FPKM	0,6199	0,3901	0,4292	3	86	2	91	42
Cutadapt_STAR _Stringtie_FPKM	0,6199	0,3901	0,4292	3	86	2	91	43
Cutadapt_STAR _Stringtie_TPM	0,6199	0,3901	0,4292	3	86	2	91	44
Cutadapt_TopHat2 _Stringtie_TPM	0,6199	0,3901	0,4292	3	86	2	91	45
Trimmomatic_hisat2 _HTSeq_IN_Raw	0,6199	0,4292	0,4077	3	38	54	95	46
Cutadapt_hisat2 _HTSeq_U_RLE	0,5931	0,4248	0,4292	29	66	2	97	47
Cutadapt_hisat2 _Stringtie_Coverage	0,5716	0,4479	0,4479	76	21	1	98	48
BBduk_STAR _Stringtie_FPKM	0,5931	0,42	0,4292	29	71	2	102	49
Trimmomatic_hisat2 _HTSeq_IN_RLE	0,5485	0,4479	0,4292	79	21	2	102	50

Continued on next page

Pipeline	RF	SVM.G	SVM.L	Rank RF	Rank SVM.G	Rank SVM.L	Sum Rank	Rank
BBduk_hisat2 _Stringtie_Coverage	0,5931	0,4479	0,4077	29	21	54	104	51
BBduk_STAR _Stringtie_Coverage	0,5931	0,4479	0,4077	29	21	54	104	52
Cutadapt_STAR _Stringtie_Coverage	0,5931	0,4479	0,4077	29	21	54	104	53
Trimmomatic_hisat2 _Stringtie_Coverage	0,5931	0,4479	0,4077	29	21	54	104	54
Trimmomatic_STAR _HTSeq_IN_RLE	0,5729	0,4292	0,4292	64	38	2	104	55
Trimmomatic_STAR _HTSeq_IN_TMM	0,5729	0,4292	0,4292	64	38	2	104	56
Trimmomatic_STAR _Stringtie_Coverage	0,5931	0,4479	0,4077	29	21	54	104	57
Trimmomatic_hisat2 _HTSeq_U_TMM	0,5723	0,4292	0,4292	71	38	2	111	58
Trimmomatic_STAR _HTSeq_U_RLE	0,5485	0,4292	0,4292	79	38	2	119	59
BBduk_hisat2 _HTSeq_IN_Raw	0,5931	0,4292	0,4077	29	38	54	121	60
BBduk_hisat2 _HTSeq_U_Raw	0,5931	0,4292	0,4077	29	38	54	121	61
BBduk_STAR _HTSeq_IN_Raw	0,5931	0,4292	0,4077	29	38	54	121	62
BBduk_STAR _HTSeq_U_Raw	0,5931	0,4292	0,4077	29	38	54	121	63
BBduk_TopHat2 _HTSeq_IN_Raw	0,5931	0,4292	0,4077	29	38	54	121	64
BBduk_TopHat2 _HTSeq_U_Raw	0,5931	0,4292	0,4077	29	38	54	121	65
Cutadapt_STAR _HTSeq_U_Raw	0,5931	0,4292	0,4077	29	38	54	121	66
Cutadapt_STAR _HTSeq_IN_Raw	0,5931	0,4292	0,4077	29	38	54	121	67
Cutadapt_TopHat2 _HTSeq_IN_Raw	0,5931	0,4292	0,4077	29	38	54	121	68
Cutadapt_TopHat2 _HTSeq_U_Raw	0,5931	0,4292	0,4077	29	38	54	121	69

Continued on next page

Pipeline	RF	SVM.G	SVM.L	Rank RF	Rank SVM.G	Rank SVM.L	Sum Rank	Rank
Trimmomatic_hisat2 _HTSeq_U_Raw	0,5931	0,4292	0,4077	29	38	54	121	70
Trimmomatic_STAR _HTSeq_IN_Raw	0,5931	0,4292	0,4077	29	38	54	121	71
Trimmomatic_STAR _HTSeq_U_Raw	0,5931	0,4292	0,4077	29	38	54	121	72
Trimmomatic_TopHat2 _HTSeq_IN_Raw	0,5931	0,4292	0,4077	29	38	54	121	73
Trimmomatic_TopHat2 _HTSeq_U_Raw	0,5931	0,4292	0,4077	29	38	54	121	74
Trimmomatic_STAR _Cufflinks_FPKM	0,6199	0,4292	0,3986	3	38	83	124	75
BBduk_STAR _HTSeq_IN_TMM	0,5283	0,4495	0,4077	89	1	54	144	76
BBduk_STAR _HTSeq_U_RLE	0,5283	0,4495	0,4077	89	1	54	144	77
Cutadapt_hisat2 _HTSeq_U_TMM	0,5485	0,4248	0,4292	79	66	2	147	78
Trimmomatic_TopHat2 _Cufflinks_FPKM	0,6199	0,4233	0,4049	3	69	82	154	79
Bbduk_hisat2 _Cufflinks_FPKM	0,6199	0,42	0,3986	3	71	83	157	80
Cutadapt_STAR _Cufflinks_FPKM	0,6199	0,42	0,3738	3	71	89	163	81
Cutadapt_TopHat2 _Cufflinks_FPKM	0,6199	0,4049	0,3869	3	80	87	170	82
BBduk_STAR _Cufflinks_FPKM	0,6199	0,3901	0,3986	3	86	83	172	83
Cutadapt_TopHat2 _Stringtie_FPKM	0,5723	0,42	0,4077	71	71	54	196	84
Trimmomatic_hisat2 _Cufflinks_FPKM	0,5931	0,4049	0,3869	29	80	87	196	85
Cutadapt_hisat2 _Cufflinks_FPKM	0,5931	0,3958	0,3986	29	85	83	197	86
BBduk_TopHat2 _Cufflinks_FPKM	0,5931	0,4049	0,3429	29	80	90	199	87
Cutadapt_hisat2 _HTSeq_IN_RLE	0,5485	0,4233	0,4077	79	69	54	202	88

Continued on next page

Pipeline	RF	SVM.G	SVM.L	Rank RF	Rank SVM.G	Rank SVM.L	Sum Rank	Rank
Cutadapt_hisat2 _HTSeq_IN_Raw	0,5716	0,4049	0,4233	76	80	52	208	89
Cutadapt_hisat2 _HTSeq_U_Raw	0,5716	0,4049	0,4233	76	80	52	208	90

Supp Table 5: F1-score values for Random Forest (RF), SVM with Gaussian kernel (SVM.G), and SVM with linear kernel (SVM.L) for each pipeline on the Zhao *et al.* dataset.

Pipeline	RF	SVM.G	SVM.L	Rank RF	Rank SVM.G	Rank SVM.L	Sum Rank	Rank
Cutadapt_STAR _Stringtie_Coverage	0,75	0,4	0,6786	1	4	2	7	1
BBduck_TopHat2 _Cufflinks_FPKM	0,4	0,6786	0,6786	5	2	2	9	2
BBduck_hisat2 _HTSeq_U_RLE	0,4	0,6786	0,6786	5	2	2	9	2
Trimmomatic_TopHat2 _HTSeq_U_TMM	0,4	0,6786	0,6786	5	2	2	9	2
Cutadapt_STAR _HTSeq_IN_RLE	0,4	0,6786	0,6786	5	2	2	9	2
Cutadapt_STAR _HTSeq_IN_TMM	0,4	0,6786	0,6786	5	2	2	9	2
Cutadapt_TopHat2 _Stringtie_FPKM	0,4	0,8615	0,4	5	1	4	10	3
BBduk_TopHat2 _Stringtie_FPKM	0,4	0,4	0,8615	5	4	1	10	3
BBduk_TopHat2 _HTSeq_IN_RLE	0,4	0,5846	0,6786	5	3	2	10	3
BBduck_STAR _Stringtie_Coverage	0,6494	0,4	0,4	2	4	4	10	3
Trimmomatic_STAR _HTSeq_IN_Raw	0,5846	0,5846	0,3571	3	3	5	11	4
Trimmomatic_STAR _Stringtie_Coverage	0,6494	0,4	0,4	2	4	4	10	3

Continued on next page

Pipeline	RF	SVM.G	SVM.L	Rank RF	Rank SVM.G	Rank SVM.L	Sum Rank	Rank
BBduck_hisat2 _Stringtie_Coverage	0,6494	0,4	0,4	2	4	4		3
Cutadapt_hisat2 _Stringtie_Coverage	0,6494	0,4	0,4	2	4	4	10	3
Cutadapt_TopHat2 _HTSeq_IN_Raw	0,3571	0,3571	0,6786	6	5	2	14/13	6
BBduk_TopHat2 _Stringtie_Coverage	0,6494	0,4	0,4	2	4	4	10	3
Trimmomatic_hisat2 _Stringtie_Coverage	0,6494	0,4	0,4	2	4	4	10	3
Cutadapt_TopHat2 _HTSeq_U_RLE	0,4	0,4	0,6786	5	4	2	11	4
Cutadapt_TopHat2 _HTSeq_U_TMM	0,4	0,6786	0,4	5	2	4	11	4
BBduck_TopHat2 _HTSeq_U_TMM	0,4	0,4	0,6786	5	4	2	11	4
Trimmomatic_hisat2 _HTSeq_U_TMM	0,4	0,6786	0,4	5	2	4	11	4
BBduck_hisat2 _HTSeq_IN_RLE	0,4	0,4	0,6786	5	4	2	11	4
BBduck_hisat2 _HTSeq_U_TMM	0,4	0,6786	0,4	5	2	4	11	4
BBduck_STAR _HTSeq_U_RLE	0,4	0,4	0,6786	5	4	2	2	11
BBduk_STAR _HTSeq_U_TMM	0,4	0,4	0,6786	5	4	2	11	4
Cutadapt_STAR _HTSeq_U_RLE	0,4	0,4	0,6786	5	4	2	11	4
Cutadapt_STAR _HTSeq_U_TMM	0,4	0,4	0,6786	5	4	2	11	4
BBduk_STAR _HTSeq_IN_RLE	0,4	0,6786	0,4	5	2	4	11	4
Trimmomatic_TopHat2 _HTSeq_U_RLE	0,4	0,4	0,6786	5	4	2	11	4
BBduk_STAR _Cufflinks_FPKM	0,4	0,4	0,6786	5	4	2	11	4
Cutadapt_hisat2 _HTSeq_U_TMM	0,4	0,6786	0,4	5	2	4	11	4

Continued on next page

Pipeline	RF	SVM.G	SVM.L	Rank RF	Rank SVM.G	Rank SVM.L	Sum Rank	Rank
BBduck_TopHat2 _HTSeq_IN_RLE	0,4	0,4	0,6786	5	4	2	11	4
Trimmomatic_hisat2 _HTSeq_IN_TMM	0,4	0,4	0,6786	5	4	2	11	4
Trimmomatic_STAR _Cufflinks_FPKM	0,4	0,4	0,6786	5	4	2	11	4
Cutadapt_hisat2 _HTSeq_IN_TMM	0,4	0,6786	0,4	5	2	4	11	3
Cutadapt_hisat2 _HTSeq_IN_RLE	0,4	0,6786	0,4	5	2	4	11	3
Cutadapt_TopHat2 _HTSeq_IN_TMM	0,4	0,4	0,6786	5	4	2	11	4
Cutadapt_TopHat2 _Stringtie_Coverage	0,5846	0,4	0,4	3	4	4	11	4
Trimmomatic_STAR _HTSeq_U_Raw	0,3571	0,3571	0,4	6	5	4	15	8
BBduck_TopHat2 _HTSeq_U_Raw	0,3571	0,4	0,4	6	4	4	14	7
BBduck_STAR _HTSeq_U_Raw	0,3571	0,4	0,4	6	4	4	14	7
Trimmomatic_hisat2 _HTSeq_U_Raw	0,3571	0,4	0,4	6	4	4	14	7
BBduck_hisat2 _HTSeq_U_Raw	0,3571	0,4	0,4	6	4	4	14	7
Cutadapt_STAR _HTSeq_U_Raw	0,3571	0,4	0,4	6	4	4	14	7
Cutadapt_hisat2 _HTSeq_U_Raw	0,3571	0,4	0,4	6	4	4	14	7
BBduck_STAR _HTSeq_IN_TMM	0,4	0,4	0,5846	5	4	3	12	5
Trimmomatic_TopHat2 _HTSeq_U_Raw	0,3571	0,4	0,4	6	4	4	14	7
Trimmomatic_hisat2 _HTSeq_IN_RLE	0,4	0,4	0,5846	5	4	3	12	5
BBduk_TopHat2 _HTSeq_IN_TMM	0,4	0,4	0,5846	5	4	3	12	5
Cutadapt_STAR _HTSeq_IN_Raw	0,3571	0,4	0,4	6	4	4	14	7

Continued on next page

**Pipeline**	**RF**	**SVM.G**	**SVM.L**	**Rank RF**	**Rank SVM.G**	**Rank SVM.L**	**Sum Rank**	**Rank**
Cutadapt_hisat2 _Cufflinks_FPKM	0,4	0,4	0,5846	5	4	3	12	5
Cutadapt_hisat2 _HTSeq_IN_Raw	0,3571	0,4	0,4	6	4	4	14	7
Cutadapt_TopHat2 _HTSeq_U_Raw	0,5	0,4	0,4	4	4	4	12	5
Trimmomatic_TopHat2 _HTSeq_IN_Raw	0,5	0,4	0,4	4	4	4	12	5
Trimmomatic_TopHat2 _Stringtie_Coverage	0,5	0,4	0,4	4	4	4	12	5
Trimmomatic_STAR _HTSeq_U_RLE	0,4	0,3571	0,4	5	5	4	14	7
Trimmomatic_STAR _HTSeq_U_TMM	0,4	0,4	0,4	5	4	4	13	6
BBduck_TopHat2 _HTSeq_U_RLE	0,4	0,4	0,4	5	4	4	13	6
Cutadapt_TopHat2 _Cufflinks_FPKM	0,4	0,4	0,4	5	4	4	13	6
Trimmomatic_hisat2 _HTSeq_U_RLE	0,4	0,4	0,4	5	4	4	13	6
BBduck_STAR _Stringtie_FPKM	0,4	0,4	0,4	5	4	4	13	6
BBduk_STAR _Stringtie_TPM	0,4	0,4	0,4	5	4	4	13	6
Cutadapt_STAR _Stringtie_FPKM	0,4	0,4	0,4	5	4	4	13	6
Trimmomatic_TopHat2 _Cufflinks_FPKM	0,4	0,4	0,3571	5	4	5	14	7
BBduk_STAR _HTSeq_IN_Raw	0,4	0,4	0,4	5	4	4	13	6
Cutadapt_STAR _Stringite_TPM	0,4	0,4	0,4	5	4	4	13	6
BBduk_hisat2 _HTSeq_IN_Raw	0,4	0,3751	0,4	5	5	4	14	7
BBduck_hisat2 _HTSeq_IN_TMM	0,4	0,4	0,4	5	4	4	13	6
Cutadapt_TopHat2 _Stringtie_TPM	0,4	0,4	0,4	5	4	4	13	6

Continued on next page

**Pipeline**	**RF**	**SVM.G**	**SVM.L**	**Rank RF**	**Rank SVM.G**	**Rank SVM.L**	**Sum Rank**	**Rank**
Trimmomatic_STAR _Stringtie_TPM	0,4	0,4	0,4	5	4	4	13	6
Trimmomatic_TopHat2 _HTSeq_IN_TMM	0,4	0,3571	0,4	5	5	4	14	7
BBduck_TopHat2 _HTSeq_IN_Raw	0,4	0,3571	0,4	5	5	4	14	7
Cutadapt_hisat2 _HTSeq_U_RLE	0,4	0,4	0,4	5	4	4	13	6
Trimmomatic_STAR _HTSeq_IN_RLE	0,4	0,4	0,4	5	4	4	13	6
Trimmomatic_STAR _HTSeq_IN_TMM	0,4	0,4	0,4	5	4	4	13	6
BBduck_hisat2 _Stringite_FPKM	0,4	0,4	0,4	5	4	4	13	6
BBduck_hisat2 _Stringite_TPM	0,4	0,4	0,4	5	4	4	13	6
Trimmomatic_hisat2 _HTSeq_IN_Raw	0,4	0,4	0,4	5	4	4	13	6
Trimmomatic_STAR _Stringite_FPKM	0,4	0,4	0,4	5	4	4	13	6
BBduck_hisat2 _Cufflinks_FPKM	0,4	0,4	0,3571	5	4	5	14	7
Cutadapt_STAR _Cufflinks_FPKM	0,4	0,3571	0,3571	5	5	5	15	8
Trimmomatic_hisat2 _Cufflinks_FPKM	0,4	0,3571	0,4	5	5	4	14	7
Trimmomatic_TopHat2 _Stringtie_FPKM	0,4	0,4	0,4	5	4	4	13	6
Cutadapt_hisat2 _Stringtie_FPKM	0,4	0,4	0,4	5	4	4	13	6
Cutadapt_hisat2 _Stringtie_TPM	0,4	0,4	0,4	5	4	4	13	6
Trimmomatic_TopHat2 _Stringtie_TPM	0,4	0,4	0,4	5	4	4	13	6
Cutadapt_hisat2 _HTSeq_IN_RLE	0,4	0,4	0,4	5	4	4	13	6
Trimmomatic_hisat2 _Stringtie_FPKM	0,4	0,4	0,4	5	4	4	13	6

Continued on next page

Pipeline	RF	SVM.G	SVM.L	Rank RF	Rank SVM.G	Rank SVM.L	Sum Rank	Rank
Trimmomatic_hisat2 _Stringtie_TPM	0,4	0,4	0,4	5	4	4	13	6
BBduck_TopHat2 _Stringtie_TPM	0,4	0,4	0,4	5	4	4	13	6

Supp Table 6: NMI-score values for Random Forest (RF), SVM with Gaussian kernel (SVM.G), and SVM with linear kernel (SVM.L) for each pipeline on the Zhao *et al.* dataset.

Pipeline	RF	SVM.G	SVM.L	Rank RF	Rank SVM.G	Rank SVM.L	Sum Rank	Rank
Cutadapt_STAR _Stringtie_Coverage	0,1984	0	0,2774	1	4	2	7	1
BBduck_TopHat2 _Cufflinks_FPKM	0	0,2774	0,2774	5	2	2	9	2
BBduck_hisat2 _HTSeq_U_RLE	0	0,2774	0,2774	5	2	2	9	2
Trimmomatic_TopHat2 _HTSeq_U_TMM	0	0,2774	0,2774	5	2	2	9	2
Cutadapt_STAR _HTSeq_IN_RLE	0	0,2774	0,2774	5	2	2	9	2
Cutadapt_STAR _HTSeq_IN_TMM	0	0,2774	0,2774	5	2	2	9	2
Cutadapt_TopHat2 _Stringtie_FPKM	0	0,5446	0	5	1	4	10	3
BBduk_TopHat2 _Stringtie_FPKM	0	0	0,5446	5	3	1	10	3
BBduk_TopHat2 _HTSeq_IN_RLE	0	0,0294	0,2774	5	3	2	10	3
BBduck_STAR _Stringtie_Coverage	0,0762	0	0	3	4	4	11	4
Trimmomatic_STAR _HTSeq_IN_Raw	0,0294	0,0294	0,0984	4	3	3	10	3
Trimmomatic_STAR _Stringtie_Coverage	0,0762	0	0	3	4	4	11	4

Continued on next page

Pipeline	RF	SVM.G	SVM.L	Rank RF	Rank SVM.G	Rank SVM.L	Sum Rank	Rank
BBduck_hisat2 _Stringtie_Coverage	0,0762	0	0	3	4	4	11	4
Cutadapt_hisat2 _Stringtie_Coverage	0,0762	0	0	3	4	4	11	4
Cutadapt_TopHat2 _HTSeq_IN_Raw	0,0984	0,0984	0,2774	2	3	2	7	1
BBduk_TopHat2 _Stringtie_Coverage	0,0762	0	0	3	4	4	11	4
Trimmomatic_hisat2 _Stringtie_Coverage	0,0762	0	0	3	4	4	11	4
Cutadapt_TopHat2 _HTSeq_U_RLE	0	0	0,2774	5	4	2	11	4
Cutadapt_TopHat2 _HTSeq_U_TMM	0	0,2774	0	5	2	4	11	4
BBduck_TopHat2 _HTSeq_U_TMM	0	0	0,2774	5	4	2	11	4
Trimmomatic_hisat2 _HTSeq_U_TMM	0	0,2774	0	5	2	4	11	4
BBduck_hisat2 _HTSeq_IN_RLE	0	0	0,2774	5	4	2	11	4
BBduck_hisat2 _HTSeq_U_TMM	0	0,2774	0	5	2	4	11	4
BBduck_STAR _HTSeq_U_RLE	0	0	0,2774	5	4	2	11	4
BBduk_STAR _HTSeq_U_TMM	0	0	0,2774	5	4	2	11	4
Cutadapt_STAR _HTSeq_U_RLE	0	0	0,2774	5	4	2	11	4
Cutadapt_STAR _HTSeq_U_TMM	0	0	0,2774	5	4	2	11	4
BBduk_STAR _HTSeq_IN_RLE	0	0,2774	0	5	2	4	11	4
Trimmomatic_TopHat2 _HTSeq_U_RLE	0	0	0,2774	5	4	2	11	4
BBduk_STAR _Cufflinks_FPKM	0	0	0,2774	5	4	2	11	4
Cutadapt_hisat2 _HTSeq_U_TMM	0	0,2774	0	5	2	4	11	4

Continued on next page

**Pipeline**	**RF**	**SVM.G**	**SVM.L**	**Rank RF**	**Rank SVM.G**	**Rank SVM.L**	**Sum Rank**	**Rank**
BBduck_TopHat2 _HTSeq_IN_RLE	0	0	0,2774	5	4	2	11	4
Trimmomatic_hisat2 _HTSeq_IN_TMM	0	0	0,2774	5	4	2	11	4
Trimmomatic_STAR _Cufflinks_FPKM	0	0	0,2774	5	4	2	11	4
Cutadapt_hisat2 _HTSeq_IN_TMM	0	0,2774	0	5	2	4	11	4
Cutadapt_hisat2 _HTSeq_IN_RLE	0	0,2774	0	5	2	4	11	4
Cutadapt_TopHat2 _HTSeq_IN_TMM	0	0	0,2774	5	4	2	11	4
Cutadapt_TopHat2 _Stringtie_Coverage	0,0294	0	0	4	4	4	12	5
Trimmomatic_STAR _HTSeq_U_Raw	0,0984	0,0984	0	2	3	4	9	2
BBduck_TopHat2 _HTSeq_U_Raw	0,0984	0	0	2	4	4	10	3
BBduck_STAR _HTSeq_U_Raw	0,0984	0	0	2	4	4	10	3
Trimmomatic_hisat2 _HTSeq_U_Raw	0,0984	0	0	2	4	4	10	3
BBduck_hisat2 _HTSeq_U_Raw	0,0984	0	0	2	4	4	10	3
Cutadapt_STAR _HTSeq_U_Raw	0,0984	0	0	2	4	4	10	3
Cutadapt_hisat2 _HTSeq_U_Raw	0,0984	0	0	2	4	4	10	3
BBduck_STAR _HTSeq_IN_TMM	0	0	0,0294	5	4	3	12	5
Trimmomatic_TopHat2 _HTSeq_U_Raw	0,0984	0	0	2	4	4	10	3
Trimmomatic_hisat2 _HTSeq_IN_RLE	0	0	0,0294	5	4	3	12	5
BBduk_TopHat2 _HTSeq_IN_TMM	0	0	0,0294	5	4	3	12	5
Cutadapt_STAR _HTSeq_IN_Raw	0,0984	0	0	2	4	4	10	3

Continued on next page

**Pipeline**	**RF**	**SVM.G**	**SVM.L**	**Rank RF**	**Rank SVM.G**	**Rank SVM.L**	**Sum Rank**	**Rank**
Cutadapt_hisat2_Cufflinks_FPKM	0	0	0,0294	5	4	3	12	5
Cutadapt_hisat2_HTSeq_IN_Raw	0,0984	0	0	2	4	4	10	3
Cutadapt_TopHat2_HTSeq_U_Raw	0	0	0	5	4	4	13	6
Trimmomatic_TopHat2_HTSeq_IN_Raw	0	0	0	5	4	4	13	6
Trimmomatic_TopHat2_Stringtie_Coverage	0	0	0	5	4	4	13	6
Trimmomatic_STAR_HTSeq_U_RLE	0	0,0984	0	5	3	4	12	5
Trimmomatic_STAR_HTSeq_U_TMM	0	0	0	5	4	4	13	6
BBduck_TopHat2_HTSeq_U_RLE	0	0	0	5	4	4	13	6
Cutadapt_TopHat2_Cufflinks_FPKM	0	0	0	5	4	4	13	6
Trimmomatic_hisat2_HTSeq_U_RLE	0	0	0	5	4	4	13	6
BBduck_STAR_Stringtie_FPKM	0	0	0	5	4	4	13	6
BBduk_STAR_Stringtie_TPM	0	0	0	5	4	4	13	6
Cutadapt_STAR_Stringtie_FPKM	0	0	0	5	4	4	13	6
Trimmomatic_TopHat2_Cufflinks_FPKM	0	0	0,0984	5	4	3	12	5
BBduk_STAR_HTSeq_IN_Raw	0	0	0	5	4	4	13	6
Cutadapt_STAR_Stringite_TPM	0	0	0	5	4	4	13	6
BBduk_hisat2_HTSeq_IN_Raw	0	0,0984	0	5	3	4	12	5
BBduck_hisat2_HTSeq_IN_TMM	0	0	0	5	4	4	13	6
Cutadapt_TopHat2_Stringtie_TPM	0	0	0	5	4	4	13	6

Continued on next page

**Pipeline**	**RF**	**SVM.G**	**SVM.L**	**Rank RF**	**Rank SVM.G**	**Rank SVM.L**	**Sum Rank**	**Rank**
Trimmomatic_STAR _Stringtie_TPM	0	0	0	5	4	4	13	6
Trimmomatic_TopHat2 _HTSeq_IN_TMM	0	0	0	5	4	4	13	6
BBduck_TopHat2 _HTSeq_IN_Raw	0	0,0984	0	5	3	4	12	5
Cutadapt_hisat2 _HTSeq_U_RLE	0	0	0	5	4	4	13	6
Trimmomatic_STAR _HTSeq_IN_RLE	0	0	0	5	4	4	13	6
Trimmomatic_STAR _HTSeq_IN_TMM	0	0	0	5	4	4	13	6
BBduck_hisat2 _Stringite_FPKM	0	0	0	5	4	4	13	6
BBduck_hisat2 _Stringite_TPM	0	0	0	5	4	4	13	6
Trimmomatic_hisat2 _HTSeq_IN_Raw	0	0	0	5	4	4	13	6
Trimmomatic_STAR _Stringite_FPKM	0	0	0	5	4	4	13	6
BBduck_hisat2 _Cufflinks_FPKM	0	0	0,0984	5	4	3	12	5
Cutadapt_STAR _Cufflinks_FPKM	0	0,0984	0,0984	5	3	3	11	4
Trimmomatic_hisat2 _Cufflinks_FPKM	0	0,0984	0	5	3	4	12	5
Trimmomatic_TopHat2 _Stringtie_FPKM	0	0	0	5	4	4	13	6
Cutadapt_hisat2 _Stringtie_FPKM	0	0	0	5	4	4	13	6
Cutadapt_hisat2 _Stringtie_TPM	0	0	0	5	4	4	13	6
Trimmomatic_TopHat2 _Stringtie_TPM	0	0	0	5	4	4	13	6
Cutadapt_hisat2 _HTSeq_IN_RLE	0	0	0	5	4	4	13	6
Trimmomatic_hisat2 _Stringtie_FPKM	0	0	0	5	4	4	13	6

Continued on next page

Pipeline	RF	SVM.G	SVM.L	Rank RF	Rank SVM.G	Rank SVM.L	Sum Rank	Rank
Trimmomatic_hisat2 _Stringtie_TPM	0	0	0	5	4	4	13	6
BBduck_TopHat2 _Stringtie_TPM	0	0	0	5	4	4	13	6

Supp Table 7: qRT-PCR expression values for the panel of genes across all samples.

Gene/Sample	49	50	51	52	53	54	55	56
Tnfaip6	9.69	10.47	10.75	10.34	10.69	10.78	10.42	10.69
Il6	10.50	10.69	10.22	11.50	10.43	10.76	9.39	8.64
Tab3	7.13	7.10	6.95	7.32	7.15	7.09	6.76	7.16
Il1b	11.83	11.95	11.44	12.11	11.62	12.07	12.17	11.66
Il18	13.44	NA	15.26	14.19	15.27	13.53	13.35	NA
Ccl2	10.80	12.17	11.39	11.56	11.93	10.85	10.26	10.89
Ccl5	11.08	10.95	10.86	10.28	10.76	10.31	9.76	10.47
Sod1	4.54	4.42	4.25	3.90	4.10	3.58	3.99	3.74
Sod2	5.10	5.07	4.82	6.32	5.34	4.84	5.07	5.41
Cat	5.21	5.39	5.32	5.67	5.27	5.43	5.08	5.30
Nrbf2	6.86	7.03	6.58	7.10	6.84	6.92	6.32	6.67
Hmox1	13.33	12.83	12.45	13.07	12.70	12.83	12.61	12.98
Gstm1	3.50	3.73	3.59	3.71	3.42	3.23	3.21	3.58
Map1lc3b	9.16	7.91	7.77	8.37	8.89	8.49	9.69	7.12
Bcl2	8.72	8.75	8.26	9.18	8.46	8.71	8.26	8.57
Bax	6.32	6.07	6.16	6.16	5.91	5.88	6.34	6.44
Casp3	6.50	6.37	6.42	6.44	6.26	6.34	5.85	6.31
Casp9	6.73	6.38	6.37	6.49	6.42	6.74	6.64	6.68
Ascl1	6.88	6.54	6.42	7.03	7.20	7.29	7.38	7.44
Wnt3a	9.98	10.23	9.83	10.40	10.71	6.43	5.66	5.28
Neurod1	4.72	4.78	5.19	5.64	6.30	5.28	5.07	5.22
Gap43	0.63	0.51	0.01	0.53	1.25	0.61	0.53	1.01
Notch1	11.49	9.81	11.54	12.30	11.35	11.37	11.17	10.75

References

S. Anders and W. Huber. Differential expression analysis for sequence count data. *Genome Biology*, 11(10):R106, 2010. doi: 10.1038/npre.2010.4282.1.

S. Anders, P. T. Pyl, and W. Huber. Htseq—a python framework to work with high-throughput sequencing data. *Bioinformatics*, 31(2):166–169, 2015. doi: 10.1093/bioinformatics/btu638.

A. M. Bolger, M. Lohse, and B. Usadel. Trimmomatic: a flexible trimmer for illumina sequence data. *Bioinformatics*, 30(15):2114–2120, 2014. doi: 10.1093/bioinformatics/btu170.

L. Breiman. Random forests. *Machine Learning*, 45(1):5–32, 2001. doi: 10.1023/A:1010933404324.

J. Brown, M. Pirrung, and L. A. McCue. Fqc dashboard: integrates fastqc results into a web-based, interactive, and extensible fastq quality control tool. *Bioinformatics*, 33(19):3137–3139, 2017. doi: 10.1093/bioinformatics/btx373.

L. A. Corchete, E. A. Rojas, D. Alonso-López, J. De Las Rivas, N. C. Gutiérrez, and F. J. Burguillo. Systematic comparison and assessment of rna-seq procedures for gene expression quantitative analysis. *Scientific Reports*, 10(1):19737, 2020. doi: 10.1038/s41598-020-76881-x.

A. Dobin et al. Star: ultrafast universal rna-seq aligner. *Bioinformatics*, 29(1):15–21, 2013. doi: 10.1093/bioinformatics/bts635.

N. N. Kachouie and M. Shutaywi. Weighted mutual information for aggregated kernel clustering. *Entropy*, 22(3), 2020. ISSN 1099-4300. doi: 10.3390/e22030351. URL `https://www.mdpi.com/1099-4300/22/3/351`.

D. Kim, G. Pertea, C. Trapnell, H. Pimentel, R. Kelley, and S. L. Salzberg. Tophat2: accurate alignment of transcriptomes in the presence of insertions, deletions and gene fusions. *Genome Biology*, 14(4):R36, 2013. doi: 10.1186/gb-2013-14-4-r36.

D. Kim, B. Langmead, and S. L. Salzberg. Hisat: a fast spliced aligner with low memory requirements. *Nature Methods*, 12(4):357–360, 2015. doi: 10.1038/nmeth.3317.

R. Kohavi. A study of cross-validation and bootstrap for accuracy estimation and model selection. In *International Joint Conference on Artificial Intelligence (IJCAI)*, volume 14, pages 1137–1145, 1995. URL `http://robotics.stanford.edu/~ronnyk/`.

B. Langmead, C. Trapnell, M. Pop, and S. L. Salzberg. Ultrafast and memory-efficient alignment of short dna sequences to the human genome. *Genome Biology*, 10(3):R25, 2009. doi: 10.1186/gb-2009-10-3-r25.

L. Macé, C. Brizais, F. Bachelot, A. Manoury, S. Thomé, C. Gloaguen, I. Garali, V. Magneron, V. Monceau, A. Sache, F. Voyer, C. Elie, L. Roy, F. Gensdarmes, M. L. B. Dmitry Klokov, and C. Ibanez. Exposure to tungsten particles via inhalation triggers early toxicity marker expression in the rat brain. *Inhalation Toxicology*, 36(4):261–274, 2024. doi: 10.1080/08958378.2024.2349895.

M. Martin. Cutadapt removes adapter sequences from high-throughput sequencing reads. *EMBnet.journal*, 17(1):10–12, 2011. doi: 10.14806/ej.17.1.200.

M. Pertea, G. M. Pertea, C. M. Antonescu, T.-C. Chang, J. T. Mendell, and S. L. Salzberg. Stringtie enables improved reconstruction of a transcriptome from rna-seq reads. *Nature Biotechnology*, 33(3):290–295, 2015. doi: 10.1038/nbt.3122.

W. Revelle. Package 'psych'. *The Comprehensive R Archive Network*, 337(338):161–165, 2015. URL `https://cran.rstudio.org/web/packages/psych/psych.pdf`.

M. D. Robinson and A. Oshlack. A scaling normalization method for differential expression analysis of rna-seq data. *Genome Biology*, 11:1–9, 2010. doi: 10.1186/gb-2010-11-3-r25.

C. Trapnell, B. A. Williams, G. Pertea, A. Mortazavi, G. Kwan, M. J. van Baren, S. L. Salzberg, B. J. Wold, and L. Pachter. Transcript assembly and quantification by rna-seq reveals unannotated transcripts and isoform switching during cell differentiation. *Nature Biotechnology*, 28(5):511–515, 2010. doi: 10.1038/nbt.1621.

G. P. Wagner, K. Kin, and V. J. Lynch. Measurement of mrna abundance using rna-seq data: Rpkm measure is inconsistent among samples. *Theory in Biosciences*, 131(4):281–285, 2012. doi: 10.1007/s12064-012-0162-3.

J. Whitham. Impact of bbduk metagenomic read trimming and decontamination. Technical report, North Carolina State University, Raleigh, NC (United States), 2021. URL `https://doi.org/10.25982/77705.1341/1779218`.
